# Design, Structural Inspection and Bio-Medicinal Applications of Some Novel Imine Metal Complexes Based on Acetylferrocene

**DOI:** 10.3390/ma15144842

**Published:** 2022-07-12

**Authors:** Mai M. Khalaf, Hany M. Abd El-Lateef, Mohamed Gouda, Fatma N. Sayed, Gehad G. Mohamed, Ahmed M. Abu-Dief

**Affiliations:** 1 Department of Chemistry, College of Science, King Faisal University, Al-Ahsa 31982, Saudi Arabia; mgoudaam@kfu.edu.sa; 2 Department of Chemistry, Faculty of Science, Sohag University, Sohag 82534, Egypt; 3 Chemistry Department, Faculty of Science, Cairo University, Giza 12613, Egypt; fatmanabilsayed85@gmail.com (F.N.S.); ggenidymohamed@sci.cu.edu.eg (G.G.M.); 4 Nanoscience Department, Basic and Applied Sciences Institute, Egypt-Japan University of Science and Technology, New Borg El Arab, Alexandria 21934, Egypt; 5 Department of Chemistry, College of Science, Taibah University, Madinah 344, Saudi Arabia

**Keywords:** acetylferrocene azomethine ligand, DFT, docking, physicochemical studies, biological activities

## Abstract

Some novel imine metal chelates with Cr^3+^, Mn^2+^, Fe^3+^, Co^2+^, Ni^2+^, Cu^2+^, Zn^2+^, and Cd^2+^ cations were produced from 2-acetylferrocene and 3-aminophenol. The new acetylferrocene azomethine ligand ((Z)-cyclopenta-1,3-dien-1-yl(2-(1-((3-hydroxyphenyl)imino)ethyl)cyclopenta-2,4-dien-1-yl)iron) and its metal ion chelates were constructed and elucidated using FT-IR, UV/Vis, ^1^HNMR, DTA/TGA, CHNClM studies, mass spectrometry and SEM analysis. According to the TGA/DTG investigation, the ferrocene moiety spontaneously disintegrates to liberate FeO. The morphology of the free acetylferrocene azomethine via SEM analysis was net-shaped with a size of 64.73 nm, which differed in Cd(II) complex to be a spongy shape with a size of 42.43 nm. The quantum chemical features of the azomethine ligand (HL) were computed, and its electronic and molecular structure was refined theoretically. The investigated acetylferrocene imine ligand behaves as bidinetate ligand towards the cations under study to form octahedral geometries in case of all complexes except in case of Zn^2+^ is tetrahedral. Various microorganisms were used to investigate the anti-pathogenic effects of the free acetylferrocene azomethine ligand and its metal chelates. Moreover, the prepared ligand and its metal complexes were tested for anticancer activity utilizing four different concentrations against the human breast cancer cell line (MCF7) and the normal melanocyte cell line (HBF4). Furthermore, the binding of 3-aminophenol, 2-acetylferrocene, HL, Mn^2+^, Cu^2+^, and Cd^2+^ metal chelates to the receptor of breast cancer mutant oxidoreductase was discovered using molecular docking (PDB ID: 3HB5).

## 1. Introduction

Most transition metal ions can easily develop stable metal chelates with azomethine molecules, which is important in inorganic chemistry. The high stability potential of azomethine metal chelates with various oxidation states allowed these compounds to be used in a variety of applications [1,2,3,4]. Because of the broad range of coordination numbers and geometries of cationic metal ions, they provide opportunities for the development of therapeutic medicines [5]. These compounds were essential to the implementation of enzymatic processes, catalysis, and magnetism, as well as materials chemistry and molecular construction [6,7]. Due to their vital structural catalytic and magnetic characteristics, as well as their pharmaceutical applications, the synthesis and characterization of such metal chelates of transition metals, such as Cu^2+^, Zn^2+^, Ni^2+^, Co^2+^ and Pd^2+^, have attracted a lot of interest recently [8].

Schiff bases have a tendency to glow, especially when their metal chelates have a rigidity planar structure and a lot of electrons [9,10]. In photometric and fluorescence investigation, Schiff base compounds have played a significant role. The salicylidene-2-aminophenol azomethine compounds, for example, have been investigated and employed as fluorimetric reagents for Mg, Al, Pd, Fe, Cr, and other cations [11,12].

Transition metal chelates of azomethine compounds with NO donors [6,13] have a great ability to possess unusual configurations, structural stability, and sensitivity to the molecular environments, so they garner particular interest [6,14]. Azomethine metal chelates were among the most known photoreactive metal chelates [15]. For example, copper and zinc are vital elements for life. Copper is associated with many copper-dependent enzymes that are key in biological processes [15,16], and zinc plays a variety of roles in the biological system, including as a component of proteins and enzymes involved in cellular signaling networks [17]. 

Ferrocene and its substitutes have grown in popularity in recent years for medical purposes and cross-linking with biomolecules such as hormones, carbohydrates, peptides, proteins, amino acids DNA, and RNA due to their stability in aqueous, aerobic media, availability of a wide range of derivatives, and electrochemical features of high quality [18,19,20,21]. As a cytotoxic or anti-aenemic agent, ferrocene has several intriguing features [22]. When cytotoxic metal compounds, such as ferrocene, were linked to polymers as prodrugs, new use and colleagues discovered that their activity was considerably increased [23,24]. Ferrocene compounds with well-known medicines have been described, including antibiotics such as cephalosporins and penicillins [18]. Moreover, structural modifications of well-known medications containing the ferrocenyl moiety, such as ferrocenyl aspirin, have been described [23,24,25] ferrocene is a combination of the anti-malarial medications chloroquine (also known as ferroquine), artemisinin, mefloquine, and quinine [18] and the anti-tumor drug tamixofen [26,27,28].

In this manuscript, we aim to prepare azomethine ligand from 2-acetylferrocene and 3-aminophenol and prepare its first-row transition metal chelates. Moreover, we will focus on studying their structure experimentally (via different spectroscopic analyses), elemental, thermal and theoretically (via DFT studies). Furthermore, some biological applications for the investigated compounds will be screened. 

## 2. Experimental

### 2.1. Reagents and Materials

All of the chemicals utilized in this study were analytical reagent grade (AR) and of the most absolute purity possible. The included 3-aminophenol and 2-acetylferrocene were received from Sigma-Aldrich, CdCl_2_, FeCl_3_·6H_2_O, CrCl_3_·6H_2_O, CoCl_2_·6H_2_O, and MnCl_2_·2H_2_O were provided from Sigma-Aldrich while ZnCl_2_, CuCl_2_·2H_2_O, and NiCl_2_·6H_2_O were provided from BDH, Merck and Strem Chemicals, respectively. EtOH (90%), Et_2_O, and DMF were utilized as organic solvents. All Cell lines used in this investigation were obtained from the American Type Culture Collection (LGC Standards, Teddington, UK). Usually, deionized water was utilized in all investigations.

### 2.2. Solutions 

All stock solutions of the prepared compounds employed in the current investigation and solutions for the anticancer study were mentioned in detail in supporting information. 

### 2.3. Instruments

All the employed instruments in the current investigation were mentioned in detail in supporting information. 

### 2.4. Molecular Structure

The Gaussian09 program suite was used to establish the ferrocene imine ligand’s molecular structure and its CoHL complex [29]. They were completely refined using the LANL2DZ basis set and the DFT-based B3LYP approach. The TDDFT approach (together with the LANL2DZ basic set) was used to predict the electronic absorption spectra of the acetylferrocene azomethine ligand to evaluate the impact of the solvent on the molecule. The molecular orbital contribution to HOMO and LUMO was also computed.

### 2.5. Molecular Docking

Molecular docking manipulations were conducted utilizing MOE2008 software, which is a stiff molecular docking software, to determine the probable binding affinity of the most active drugs against the receptor of oxidoreductase mutation in breast cancer (3HB5) [30]. Docking is a molecular graphics tool that calculates and displays possible docking modes between a receptor and 3-aminophenol, 2-acetylferrocene, free ligand, Mn^2+^, Cu^2+^, and Cd^2+^ chelates. These compounds, as well as the receptor, must be entered in PDB format. Water, co-crystallize ligands, ionization sphere (Cl), and other elements that are not supported (e.g., Na, K, Hg, etc.) were eliminated from the crystallization molecules [31]. Gaussian03 software was used to construct the structures of 2-acetylferrocene, 3-aminophenol, the free ligand, Mn^2+^, Cu^2+^, and Cd^2+^ azomethine chelates in PDB file format. The database of proteins (http://www.rcsb.org./pdb, accessed on 1 January 2000) was used to retrieve the receptor of oxidoreductase mutation in breast cancer (3HB5).

### 2.6. Synthesis of Acetylferrocene Azomethine Ligand

The acetylferrocene azomethine ligand (HL) was synthesized by refluxing a mixture of 2-acetylferrocene (21.84 mmol, 4.98 g) dissolved in EtOH and 3-aminophenol (21.84 mmol, 2.38 mL). The resultant mixture was heated and agitated for 4 h, during which time a black solid product was isolated. It was dried under a vacuum after being filtered, recrystallized, and washed with Et_2_O.

^1^HNMR of HL ligand, phenolic -OH (δ 8.78 ppm, 6.45, s, 1H, phenolic OH); aromatic proton in the free acetylferrocene azomethine (δ 5.93–6.76 ppm, 17.07, m, 4H, ArH); cyclopentadienyl (δ 4.23–4.83 ppm, 10.52, m, 9H, ferrocene) and methyl (δ 3.33 ppm, 13.98, s, 3H, CH_3_). 

### 2.7. Synthesis of Ferrocene Azomethine Metal Chelates

The Cr^3+^, Mn^2+^, Fe^3+^, Co^2+^, Ni^2+^, Cu^2+^, Zn^2+^, and Cd^2+^ chelates were synthesized in combination with a 1:1 molar mixture of hot EtOH solution (70 °C) of chloride salts of the investigated cations (1.25 mmol; 0.33 g of CrCl_3_·6H_2_O, 0.20 g of MnCl_2_·2H_2_O, 0.20 g of FeCl_3_·6H_2_O, 0.30 g of CoCl_2_·6H_2_O, 0.30 g of NiCl_2_·6H_2_O, 0.17 g of CuCl_2_·2H_2_O, 0.17 g of ZnCl_2_ and 0.23 g of CdCl_2_) and the ligand (HL) (0.4 g, 1.25 mmol). The resultant mixture was agitated for 1 h under reflux, after which the metal chelates separated. They were extracted through filtering and purified by washing with a small amount of ethanol several times followed by Et_2_O multiple times.

^1^HNMR of [Cd(HL)(H_2_O)_2_Cl_2_] complex, phenolic-OH (δ 8.71 ppm, 5.27, s, 1H, phenolic OH); aromatic proton in the free acetylferrocene azomethine (δ 5.92–6.79 ppm, 2.85, m, 4H, ArH); cyclopentadienyl (δ 4.23–4.77 ppm, 10.97, m, 9H, ferrocene) and methyl (δ 3.23 ppm, 14.97, s, 3H, CH_3_). 

### 2.8. Biological Activity

#### 2.8.1. Anti-Pathogenic Activity 

The antimicrobial activities of the compounds under investigation were screened according to the Well diffusion approach [32,33,34]. The detailed method was shown in the supporting information file.

#### 2.8.2. Optimization of Anticancer Study 

The cytotoxicity of the prepared compounds against the MCF-7 cell line was investigated according to employed methods in the literature [33,35,36]. The detailed method was shown in the supporting information file.

## 3. Results and Discussions 

### 3.1. Identification of the Acetylferrocene Azomethine Ligand and Its Metal Chelates

#### 3.1.1. Elemental Analyses and Molar Conductivity Study

Elemental analyses and some physical properties of the newly synthesized acetylferrocene azomethine ligand and its metal chelates were recorded in Table 1. The experimental data of elemental analyses were in agreement with the theoretical calculation from each compound’s empirical formula. Acetylferrocene azomethine ligand was soluble in ethanol and its metal chelates were stable, nonhygroscopic, and soluble in most organic solvents such as DMF and DMSO and insoluble in ethanol, methanol and acetone. The molar conductivity values of metal chelate showed that all metal chelates were electrolytes except Cu^2+^, Zn^2+^, and Cd^2+^ cations were non-electrolytes [37], indicating that no anions were located in the ionization sphere. The molar conductivity data for Fe^3+^ and Ni^2+^ chelates are close to those previously reported for 1:1 electrolytes [37,38]. In addition, Cr^3+^, Mn^2=^ and Co^2+^ complexes have molar conductivity values close to 1:2 electrolytes. 

#### 3.1.2. FT-IR Spectral Studies

Several trials were carried out to prepare crystals suitable for X-ray analysis using single solvent or mixed solvents but unfortunately all trials were failed. Therefore, spectroscopic tools such as IR are significant and can help in describing the structures of the complexes.

The formation of acetylferrocene azomethine ligand (HL) was indicated by the inclusion of a sharp IR band at 1655 cm^−1^ owing to υ(C=N), while no bands ascribed to υ(C=O) or to υ(NH_2_) had been recognized [1]. FT-IR spectral data of metal chelates were compared with the free acetylferrocene azomethine ligand (HL) shown in Table 2. The azomethine ligand showed a characteristic band of the OH group in the 3444 cm^−1^ regions [39]. The shift of this band to (3411–3446 cm^−1^) in the FT-IR spectra of the metal chelates (Figure S1) cannot be assigned to the cations coordinated to the ligand via its phenolic OH. This shift can be also attributed to the presence of water molecules in the complex structures which render it difficult to confirm the coordination of the phenolic group. The band found at 1277 cm^−1^ in the IR spectrum of the azomethine ligand was assigned to υ(C-O) phenolic group [40]. The existence of this band at 1267–1283 cm^−1^ in the IR spectra of the complexes can account for the participation of phenolic oxygen in binding to the metal ions [40]. The shift of the C=N band to lower frequencies (1597–1649 cm^−1^) in the vibration spectra of the metal chelates compared to the acetylferrocene azomethine ligand suggested bonding of the imine nitrogen to the investigated cations [1,41].

Two weak bands in areas of 894–980 and 815–874 cm^−1^ attributable to (H_2_O) vibrations in the swinging and twisting modes, respectively, proved the presence of coordinated aqua molecules in all the metal chelates except Zn(II) acetylferrocene chelate [38]. The presence of coordinating water molecules was also inferred by the appearance of r(H_2_O) and w(H_2_O) vibrations, which were later determined by TGA analysis [42]. 

The new (M-O) and (M-N) weak intensity bands were assigned in the regions 520–598 cm^−1^ and 450–499 cm^−1^, respectively [41]. This implies that phenolic oxygen and azomethine nitrogen were both involved in the formation of all chelates [43]. Based on the previous facts, it was determined that the acetylferrocene ligand acted as a neutral bidentate ligand as metal ions coordinated to phenolic oxygen and nitrogen of the azomethine group. 

#### 3.1.3. Mass Spectrometry

**Figure 1:**
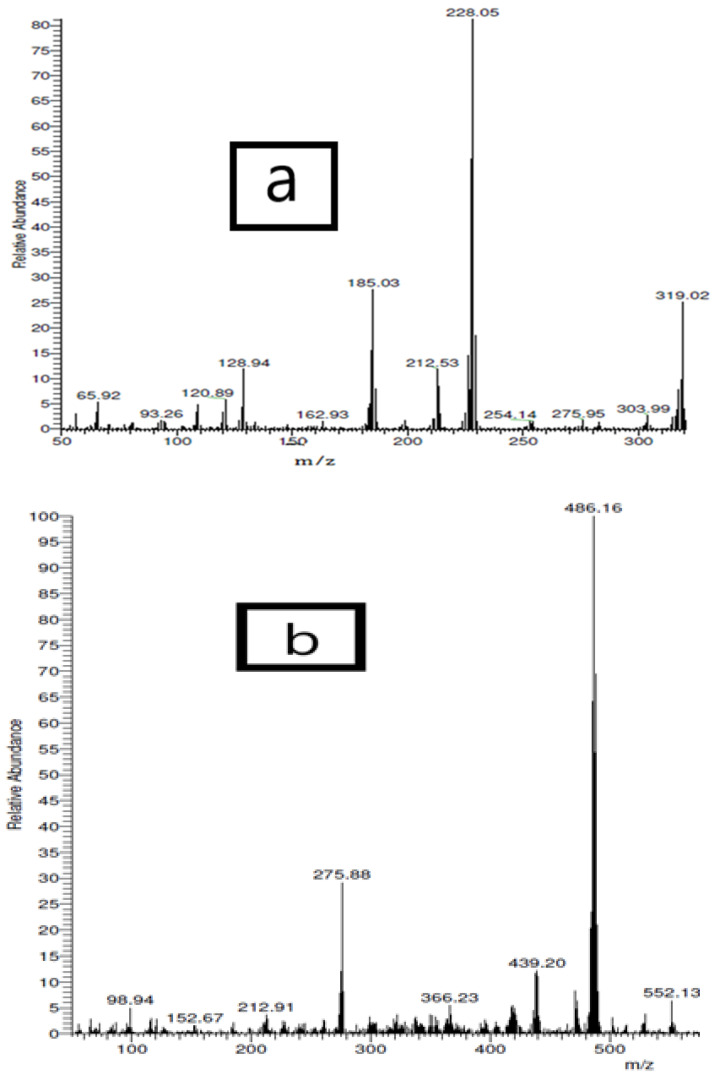
Mass spectrometry of (**a**) the free acetylferrocene azomethine ligand and (**b**) [Co(HL)(H_2_O)_4_]Cl_2_·2H_2_O.

The mass spectra of the free ligand and its Co(II) complex are represented in Figure 1. The spectrum of the free ligand showed m/z peak at 319.02 amu with a relative intensity of 25%, which corresponds to the (C_18_H_17_NOFe) molecule. By matching molecular formula weight of 319.06 amu, which was in best accordance with this molecule, the predicted molecular formula of the investigated acetylferrocene azomethine ligand was confirmed. While the spectrum of [Co(HL)(H_2_O)_4_]Cl_2_·2H_2_O complex showed molecular ion peak at 552.13 amu which is highly agreed upon, the expected molecular weight (554 g/mol) that confirms this chelate formation. 

#### 3.1.4. ^1^H-NMR Spectroscopic Studies

Proof of the bonding type of the ligand was also confirmed by the ^1^H-NMR spectra of the acetylferrocene azomethine (Figure S2a) in DMSO and deuterated solvent and its diamagnetic Cd(II) acetylferrocene chelate (Figure S2b). The titled Schiff base HL ^1^H-NMR spectrum revealed a singlet signal at the downfield value at δ 8.78 ppm (s, 1H, phenolic OH) which is attributed to phenolic -OH proton and shifted to δ 8.71 ppm (s, 1H, phenolic OH) in the Cd(II) complex. With the addition of D_2_O, the OH signal vanished, confirming its assignment [44]. The detected signals of aromatic proton in the free acetylferrocene azomethine ligand in the region of δ 5.93–6.76 ppm (m, 4H, ArH) showed a small shift in the Cd(II) complex at δ 5.92–6.79 ppm (m, 4H, ArH), as a result of chelation-induced changes in electron density [45]. Moreover, the free Schiff base showed signals as δ 4.23–4.83 ppm and δ 3.33 ppm which were attributed to cyclopentadienyl (m, 9H, ferrocene) [46] and methyl (s, 3H, CH_3_) [37] protons, respectively, that showed a slight shift to δ 4.23–4.77 ppm (m, 9H, ferrocene) and δ 3.23 ppm (s, 3H, CH_3_), respectively, in the spectrum of the Cd(II) complex. The overall results of ^1^H NMR studies confirmed the binding of metal ions to the free ligand through OH phenolic group without deprotonation, so it was concluded that our ligand was neutral bidentate with the general formula HL. The ^13^C NMR spectrum of the acetylferrocene Schiff base ligand showed peaks at δ 18.2 ppm for CH_3_, 109.4, 114.7, 114.1, 131, 150.6 and 160 ppm for aromatic carbons and 124.7, 137.3, 141 and 165.5 ppm for cyclopentadienyl carbons. Meanwhile, the ^13^C NMR spectrum of the acetylferrocene Schiff base Cd(II) complex showed peaks at δ 18.3 ppm for CH_3_, 109.5, 114.8, 113.9, 131.2, 150.5 and 160.2 ppm for aromatic carbons and 124.9, 137.4, 141.2 and 165.6 ppm for cyclopentadienyl carbons. The small shift in peak position can be attributed to change in carbon skeleton as the result of chelate formation.

#### 3.1.5. Geometrical Optimization of the Ligand and CoHL Complex

**Figure 2:**
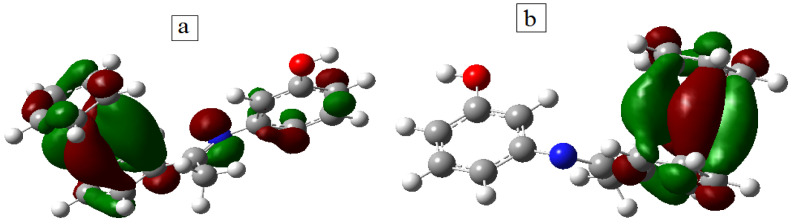

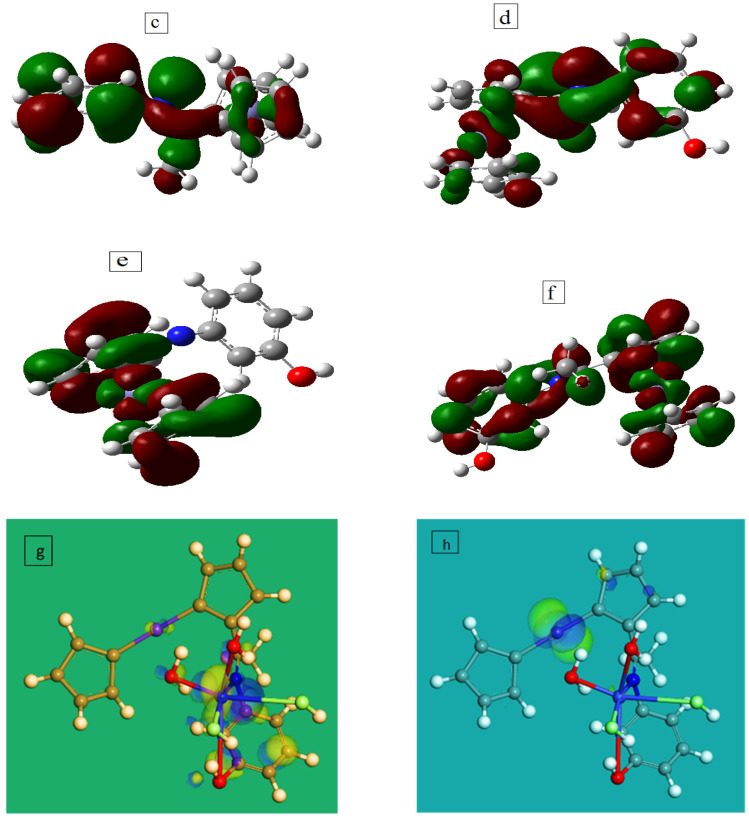
Molecular orbitals of the free acetylferrocene azomethine ligand HL (**a**) HOMO (**b**) HOMO-1 (**c**) HOMO-2 (**d**) LUMO (**e**) LUMO + 1 (**f**) LUMO + 2; and [Co(HL)(H_2_O)_4_]Cl_2_·2H_2_O complex (**g**) HOMO and (**h**) LUMO.

At the DFT level of theory, the full configuration of the free acetylferrocene azomethine ligand (HL) and its Co(II) complex was accomplished [44]. Figure S3 shows a perspective of the optimized structures of the HL and CoHL and their atom numbering. Table S1 show specified bond angles and distances of HL. The HL ligand shows distances (N2-C1) 1.30 Å, (N2-C3) 1.42 Å and (C6-O36) 1.40 Å. While in complexation, we observed stretching in these bonds to be (O22-Co) 1.71 Å, and (N7-Co) 1.59 Å (Table S2), respectively, which indicate the complexation through azomethine nitrogen and phenolic OH. Moreover, the Co(II) complex exhibited change in bond angles which confirm the complexation sites, HL shows angles C1-N2-C3 (125.78) and H37-O36-C6 (111.84). In CoHL complex, the angles which changed in Co(II) complex to be Co(21)-O(22)-C(3)56.14 and Co(21)-N(7)-C(1) 83.96 which confirming the chelation though azomethine nitrogen and phenolic OH. The major orbitals implicated in chemical consistency are the highest occupied molecular orbital (HOMO) and the lowest unoccupied molecular orbital (LUMO). The HOMO reflects the capacity to give an electron, whereas the LUMO as an electron acceptor reflects the ability to secure an electron. The HOMO and LUMO for the acetylferrocene azomethine ligand (HL) and its Co(II) complex were depicted in Figure 2. Table 3 lists the estimated quantum chemical factors. The Equations (1)–(8) below were used to derive further characteristics such as E, absolute electronegativities, chemical potentials, Pi, absolute hardness, absolute softness, global electrophilicity, global softness, S, and additional electronic charge, *N*_max_ [47,48,49,50,51].

∆*E* = *E*_LUMO_ − *E*_HOMO_ (1)

(2) $$\chi =\frac{-(E_{\mathrm{HOMO}}+E_{\mathrm{LUMO}})}{2}$$

(3) $$\eta =\frac{\left(E_{\mathrm{LUMO}}-E_{\mathrm{HOMO}}\right)}{2}$$

(4) $$\sigma =\frac{1}{\eta }$$

*P_i_* = −*χ* (5)

(6) $$S=\frac{1}{2\eta }$$

(7) $$\omega =\frac{P_{i}^{2}}{2\eta }$$

(8) $$\Delta N_{\max}=-\frac{P_{i}}{\eta }$$

These calculated parameters showed that the free ligand was able to donate electrons to metal ions chelates with lower energy difference (3.75 eV), with Δ*E* of the free ligand (4.29 eV), so the Co(II) complex had high stability and high biological activities. 

#### 3.1.6. UV–Vis Absorption Investigations

The electronic spectrums of the free acetylferrocene azomethine ligand (10^−4^ M) and its metal chelates (10^−5^ M) were recorded in (1:3 *v*/*v*) ethanol: DMF in the range of 200–700 nm. HL’s spectra comprised four major bands at 232, 268, 334, and 446 nm. The first two bands were allocated to n-π* transition of the benzene ring [3] which shifted to 233–290 nm in the metal chelates. The third band was attributed to π-π* transition of the imine group [52] which shifted to 302–366 nm in all-metal chelates except for Mn(II) and Ni(II) cation chelates, which disappeared. The fourth band was assigned to charge transfer transitions [52] from aromatic ring to imine group that disappeared in the metal chelates.

**Table 3:** The different quantum chemical parameters of the free acetylferrocene azomethine ligand.

The Calculated Quantum Chemical Parameters		
	HL	[Co(HL)(H_2_O)_4_]Cl_2_·2H_2_O
*E*_HOMO_ (eV)	−5.77	−1.84
*E*_LUMO_ (eV)	−1.48	−1.25
Δ*E* (eV)	4.29	3.75
*χ* (eV)	3.63	1.32
*η* (eV)	2.15	1.95
*σ* (eV)^−1^	0.47	0.51
*P_i_* (eV)	−3.63	−1.32
*S* (eV)^−1^	0.23	0.26
*ω* (eV)	3.06	0.45
Δ*N*_max_	1.69	0.68

#### 3.1.7. Thermal Analysis

Thermal decompositions were done under an air environment at a rate of heating 10 °C min^−1^. The thermogravimetric data for the acetylferrocene azomethine (HL) and its metal chelates were depicted in Table 4 within the range of temperature of 30 to 1000 °C (Figure S4a). The acetylferrocene azomethine ligand TG data revealed three steps of decomposition. The three stages took place between 35 and 695 °C (DTG maxima peaks temperatures at 198, 363, and 689 °C) and were associated with a loss of mass of 77.66% (loss of mass predicted = 77.31%) in agreement with the loss of the organic moiety of the ligand leaving iron(II) oxide residue. 

The [Cr(HL)(H_2_O)_3_Cl]Cl_2_·3H_2_O chelate was thermally disintegrated in two conclusive decomposition steps. The two stages reported a found mass loss to be 9.17% (loss of mass predicted = 9.22%) and 51.11% (loss of mass predicted = 50.72%) within the range of temperature of 30–140 °C (DTG peak at 77 °C) and 140–1000 °C (DTG peak at 854 °C), which may be attributed to the disposal of three hydrated water molecules, three HCl molecules, one molecule of coordinated water, and C_11_H_18_ClNO_0.5_ fragment (Figure S5b). ½Cr_2_O_3_ and FeO contaminated with carbon were left as a residue. The total reported loss of mass was 59.88% (loss of mass predicted = 60.32%). 

The TG curve of the Mn(II) chelate showed six stages of decomposition within the range of temperature 45–1000 °C. The fourth stage was corresponding to the loss of hydrated and coordinated water molecules and ammonia gas with a loss of weight equated to 18.01% (loss of mass predicted = 16.62%) within a range of temperature 45–440 °C (DTG maximum temperatures are 129, 179, 254 and 411 °C). The last two steps within the temperature range 440–1000 °C were corresponding to the loss of two HCl, ethane, and C_16_H_10_ molecules with temperature maxima at 613 and 967 °C with the reported loss of mass of 55.18% (loss of mass predicted = 56.58%) (Figure S5c). MnO and FeO were left as residues with mass percent of 26.81% (loss of mass predicted = 26.80%). 

The thermogram of Fe(III) chelate showed four decomposition steps within the range of temperature 50–1000 °C. The 1st and 2nd decomposition stages within the range of temperature 50–580 °C (T_s_ = 100 and 350 °C) correspond to the disposal of three hydrated water molecules, ammonia gas, methane gas and two molecules of HCl with a loss of mass of 29.70% (loss of mass predicted = 27.95%). The 3rd and 4th steps (580–1000 °C) matched with the elimination of part of the ligand with a loss of mass of 20.21% (loss of mass predicted = 20.35%), leaving contaminated ½Fe_2_O_3_ and FeO as residues with a mass percent of 50.09% (loss of mass predicted = 51.88%).

[Co(HL)(H_2_O)_4_]Cl_2_·2H_2_O and [Cu(HL)(H_2_O)_2_Cl_2_]·2H_2_O complexes demonstrated three stages of decomposition. The 1st decomposition stage took place within the range of temperature of 45–190 °C and 45–130 °C matched with the disposal of two hydrated water molecules and ammonia and methane gases (loss of mass found = 12.52%; loss of mass predicted = 12.40%) and two water molecules of hydration (loss of mass of 5.78%; predicted = 6.84%) for Co(II) and Cu(II) complexes, respectively. The 2nd and 3rd decomposition stages within the range of temperature of 190–1000 °C and 130–1000 °C correspond to the disposal of three coordinated water and C_13_H_12_Cl_2_ molecules and C_11_H_19_NCl_2_ and coordinated water molecules with a loss of mass of 51.94% (predicted = 52.65 %) and mass losses equated to 48.38% (predicted = 48.29%), for Co(II) and Cu(II) complexes, respectively. CoO and FeO and CuO and FeO residues with carbon contamination have remained. The cumulative amount of weight loss equates to 64.45% (predicted = 65.05%) and 54.15% (predicted = 55.13%) for Co(II) and Cu(II) complexes, respectively.

The TG curve of the Ni(II) chelate showed five stages of decomposition within the range of temperature of 35–1000 °C. The first stage at 35–145 °C (T_s_ = 81 °C) matched with the disposal of two water molecules of hydration and ammonia gas, while the second step involved loss of HCl, CH_4_, and two coordinated water molecules within a range of temperature from 145 °C to 355 °C. The last three stages took place within the range 335–1000 °C and corresponded to the disposal of the C_11_H_11_Cl molecule with a reported loss of mass of 33.97% (predicted loss of mass = 33.09%) leaving carbon contaminated NiO and FeO residues with loss of mass 40.10% (predicted = 40.696%). The cumulative amount of weight loss was 59.90% (predicted = 59.31%). 

The TG curve of Zn(II) and Cd(II) chelates revealed a peak at 77 °C and 185 °C in the range of temperatures 45–275 °C and 40–220 °C that was owing to a weight loss of 6.69% (predicted weight loss = 6.92%) and 27.68% (predicted = 27.86%), respectively. This stage could be assigned to the disposal of a hydrated water molecule and methane gas and two hydrochloride molecules, one coordinated water molecule, ammonia gas, and a C_3_H_6_ fragment for Zn(II) and Cd(II) complexes, respectively. The final three decomposition stages took place at 275–1000 °C and 220–600 °C range of temperature with Ts = 402 °C, 585 °C, and 955 °C and 236 °C, 266 °C, and 519 °C were found to have the disposal of a mass of 54.88% (predicted loss of mass = 54.53%) and 35.18% (predicted mass loss = 34.91%) corresponding to the loss of ethene, two HCl and ammonia gas molecules and loss of C_15_H_8_ fracture for Zn(II) and Cd(II) complexes, respectively (Figure S5h,i). At the end of the curves, the ZnO and FeO were loaded with atoms of carbon, and CdO and FeO were the residues, with a total loss of weight equated to 61.56% (predicted = 61.44%) and 62.86% (predicted = 62.77%) (Figure S5), for Zn(II) and Cd(II) complexes, respectively. The presence of carbon in the metallic residue was confirmed by dissolving the Al containing the residue in concentrated HCl solution where black residues were observed.

#### 3.1.8. SEM

**Figure 3:**
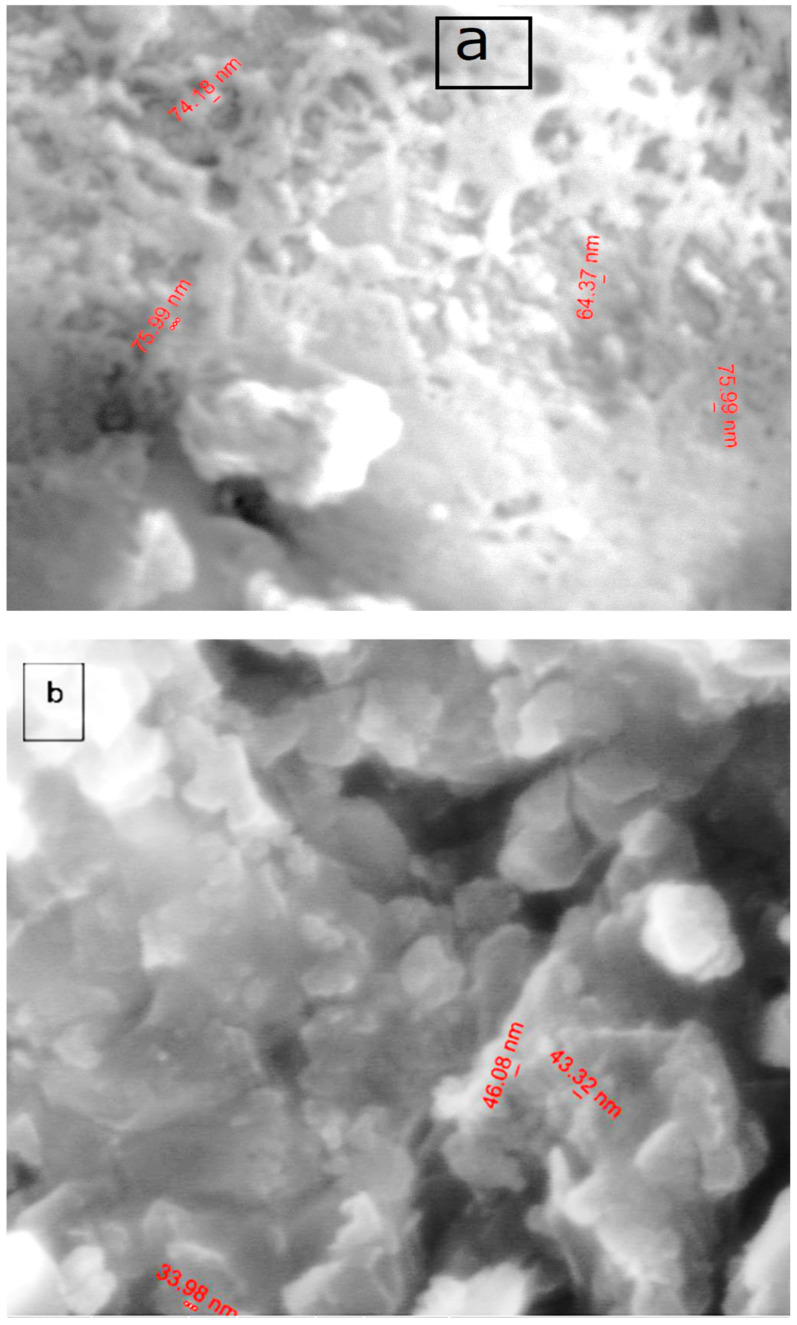
SEM graphs of (**a**) ligand and (**b**) [Cd(HL)(H_2_O)_2_Cl_2_] complex.

The surface morphology, structure, and size of the free acetylferrocene azomethine ligand and Cd(II) chelate were carried out using SEM images (Figure 3a,b). The SEM micrographs of the free ligand differed significantly from [Cd(HL)(H_2_O)_2_Cl_2_] complex owing to the coordination of metal cation to the donor sites in the free acetylferrocene azomethine ligand [53,54]. The morphology of the free acetylferrocene azomethine was net-shaped with a size of 64.73 nm which differed in Cd(II) complex to be a spongy shape with a size of 42.43 nm. SEM analysis yielded a size distribution that indicated that the acetylferrocene azomethine ligand (HL) and the Cd(II) complex were polycrystalline with nanosized grains [53,55].

#### 3.1.9. Structural Manipulation 

**Figure 4:**
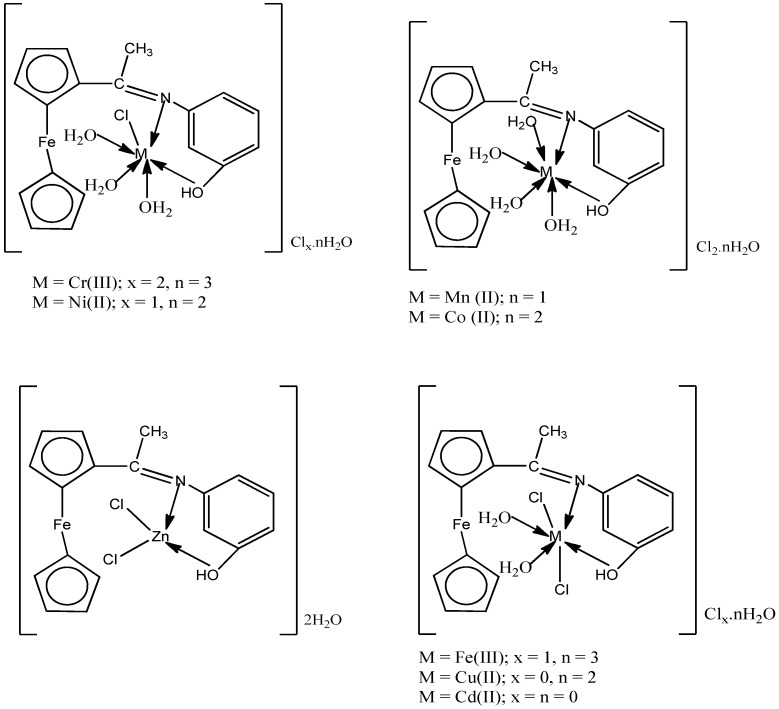
The suggested structures for acetylferrocene azomethine metal chelates.

According to the physicochemical and spectral facts supplied and addressed previously, metal chelate structures have been confirmed, and proposed structural formulas for metal chelates are illustrated in Figure 4.

### 3.2. Antimicrobial Activities

**Figure 5:**
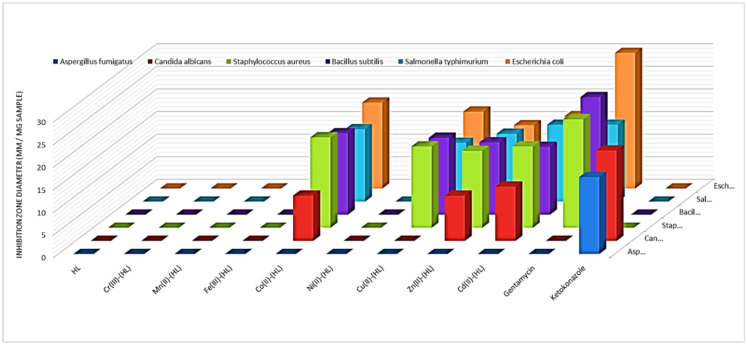
Biological activities of acetylferrocene azomethine ligand HL and its metal chelates.

The examined compound’s antibacterial activity was assessed in vitro against the microorganisms *Aspergillus fumigatus* and *Candida albicans* (fungi), *Staphylococcus aureus,* and *Bacillus subtilis* (G + ve bacteria), and *Salmonella typhimurium* and *Escherichiacoli* (G-ve bacteria) by the diffusion agar method. The inhibition zone diameter values of the tested samples were represented in Table S3 and Figure 5, indicating that the free ligand, Cr^3+^, Mn^2+^, Fe^3+^, and Ni^2+^ metal chelates had no activity against some microorganisms, while other metal chelates showed some activity against some microorganisms, demonstrating the effect of chelation as the majority of metal chelates are more active than their respective azomethine ligand. In some cases, the free acetylferrocene azomethine ligand is more active than metal chelates against bacteria. Chelation either increases or decreases antibacterial activity; it can also be neutral. As a result, metal chelation may enhance or diminish the therapeutic effectiveness of organic molecules (drugs) [56,57,58]. By further stabilizing the medication and/or lowering the biodegradability/metabolic decay of the organic ligands through chelation, the characteristic may be preserved [59]. The activity indexes of the tested compounds were calculated and plotted in Figure S5a,b according to the following equation [60,61]:

(9) $$\mathrm{Activity}\;\mathrm{index}\;(A)=\frac{\mathrm{Compound}\;\mathrm{inhibition}\;\mathrm{zone}\;(\mathrm{mm})}{\mathrm{Standard}\;\mathrm{drug}\;\mathrm{inhibition}\;\mathrm{zone}\;(\mathrm{mm})}\times 100$$

The Cd(II) complex exhibited the greatest activity index, according to the findings (100 percent). 

The discrepancies in metal chelate action against microbial species are related to variances in microbial cell ribosomes or cell impermeability. The lower activity of complexes in comparison to others could be due to low lipid solubility, which prevents the metal ion from reaching the cell wall’s preferred location of action and disrupting normal cell function. Other variables such as solubility, size, dipole moment, coordinating sites, the redox potential of metal ions, solubility, the bond length between metal and ligand, geometry of complexes, steric, pharmacokinetic, concentration, and hydrophobicity also play a role in evaluating the antibacterial activity of metal chelates [62].

### 3.3. Anticancer Activities

Chemotherapy is the most common treatment option for both localized and metastatic cancer [63]. Therefore, the investigated compounds were screened for their in vitro growth inhibitory and cytotoxicity activities against breast cancer cell line.

Antitumor efficiency of the free acetylferrocene azomethine ligand (HL) and its metal chelates was screened against breast cancer cell line (MCF7) and normal melanocyte cell line (HBF4) in high concentration (100 µg/mL). 

The cytotoxicity of the compounds studied was measured using the median antiproliferative concentration (IC_50_), which required the compounds to have a 50% cytotoxic effect on cancer cells after 48 h of exposure.

**Figure 6:**
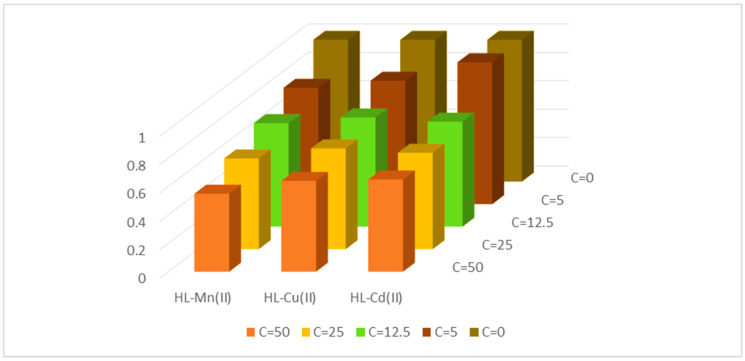
Anticancer activity of the azomethine acetylferrocene metal chelates.

Only Mn(II), Cu(II) and Cd(II) metal chelates showed an inhibitory effect >70% against the MCF7 cell line, so they were carried out in four different concentrations; the findings were shown in Table S4 and demonstrated in Figure 6. 

The order of cytotoxicity activity against the two cancer cell lines of the investigated chemicals is obvious from the data; it was Cd(II) > Cu(II) > Mn(II) complex. The highest activity of the Cd(II) complex than the others may be attributed to the function of the Cd(II) chelate as a competitive inhibitor of hemeoxygenase (HMOX1), which is produced in large amounts in solid tumors [64], in humans and animal tumor models. 

### 3.4. Molecular Docking

The uncontrolled proliferation of aberrant cells is referred to as cancer. Breast cancer and lung cancer were the most frequently diagnosed fatal cancers in women over the world, with significant mortality rates. Breast cancer is predicted to affect more than 90,000 Indian women in the next years, with over 50,000 women dying each year [63]. Computer drug design highly depends on molecular docking [65]. The objective of molecular docking is to mimic the process of molecular recognition. It is also necessary to identify an appropriate shape for both the protein and the drug, with their positions relative to one another, in order to lower the overall system’s free energy. 

**Figure 7:**
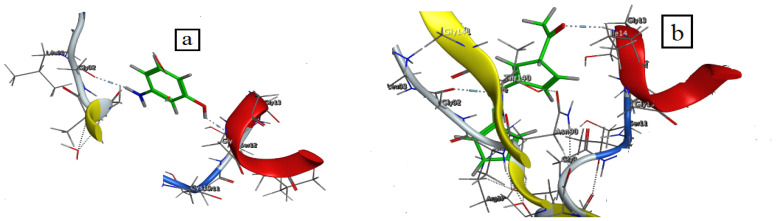

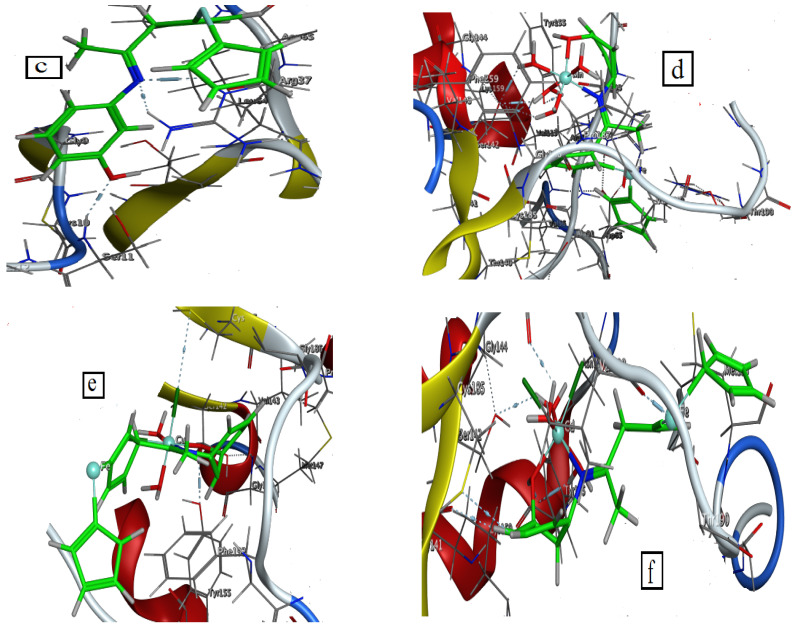
3D plot of the interaction between the receptor of 3HB5 with (**a**) 3-aminophenol, (**b**) 2-acetylferrocene, (**c**) HL, (**d**) Mn(II) complex, (**e**) Cu(II) complex and (**f**) Cd(II) complex.

The molecular docking of starting materials 3-aminophenol and 2-acetylferrocene, the free acetylferrocene azomethine ligand HL, Mn(II), Cu(II), and Cd(II) metal chelates was carried out with the receptor of breast cancer (2HB5). The docking investigation revealed a significant interaction between these drugs and the receptor (3HB5), as well as a low binding energy calculation, which was recorded in Table S5 and Figure 7 demonstrating 3D structures of possible interactions.

According to our data, it was found that binding energies can be ordered ascendingly as follows: 3-aminophenol (−3.3 kcal/mol) < 2-acetylferrocene (−6.2 kcal/mol) < HL (−10 kcal/mol) < Cu(II) complex (−20.4 kcal/mol) < Mn(II) complex (−62.3 kcal/mol) < Cd(II) complex (−73.3 kcal/mol). As shown, Cd(II) complex had the highest binding energy which matched with anticancer results that had IC_50_ 3.5 μg/mL.

## 4. Conclusions

The chemical structures of a novel free acetylferrocene azomethine ligand (HL) and the coordination chemistry of some transition metal cations—Cr^3+^, Fe^3+^, Mn^2+^, Co^2+^, Ni^2+^, Cu^2+^, Cd^2+^, and Zn^2+^ cations—with the newly synthesized ligand were elucidated using various physicochemical techniques as molar conductance proved the electrolytic nature of metal chelates by showing that all metal chelates were electrolytes, except Cu(II), Zn(II) and Cd(II) metal chelates could not conduct electricity in their solutions. IR studies showed that the free ligand acted as a neutral bidentate ligand as metal ions coordinated with phenolic oxygen and nitrogen of the imine group. ^1^HNMR analysis confirmed that complexation took place without deprotonation of the phenolic OH group. From geometrical calculations, the calculated parameters showed that the free ligand was able to donate electrons to metal ions forming stable metal chelates. From SEM studies, it was found that the free acetylferrocene azomethine ligand and its Cd(II) chelate were in nano size. Mn(II) complex only demonstrated IC_50_ value of 36.70 μg/mL. MOE investigations were employed to determine the binding orientation or conformation of 3-aminophenol, 2-acetylferrocene, HL, Mn(II), Cu(II), and Cd(II) metal chelates in the active site of the 3HB5 breast cancer receptor. Through ionic and hydrogen donor interactions with the examined protein, the [Mn(HL)(H_2_O)_4_]Cl_2_·H_2_O complex demonstrated the highest binding ability with the lowest binding energy.

## Figures and Tables

**Table 1:** Physical and analytical data of organometallic acetylferrocene azomethine ligand and its metal chelates.

Compound (Molecular Formula)	Colour (%Yield)	M.p. (°C)	% Calcd. (Found)					Λ_m_ Ω^−1^ mol^−1^ cm^2^
			C	H	N	Cl	M	
HL (C_18_H_17_NOFe)	Greenish yellow (80)	92–95	67.61 (66.20)	5.32 (5.57)	4.38 (4.58)	------	17.68 (18.18)	------
[Cr(HL)(H_2_O)_3_Cl]Cl_2_·3H_2_O (C_18_H_29_NO_6_Cl_3_CrFe)	Black (70)	>300	36.86 (37.00)	4.95 (4.39)	2.39 (2.68)	18.17 (18.78)	18.52 (18.01)	110
[Mn(HL)(H_2_O)_4_]Cl_2_·H_2_O (C_18_H_27_NO_6_Cl_2_MnFe)	Black (75)	>300	40.34 (40.65)	5.04 (4.19)	2.61 (2.52)	13.26 (14.18)	20.63 (20.14)	104
[Fe(HL)(H_2_O)_2_Cl_2_]Cl·3H_2_O (C_18_H_27_NO_6_Cl_3_Fe_2_)	Black (79)	>300	37.73 (37.56)	4.72 (3.71)	2.45 (2.57)	18.60 (18.82)	19.74 (20.62)	59
[Co(HL)(H_2_O)_4_]Cl_2_·2H_2_O (C_18_H_29_NO_7_Cl_2_CoFe)	Black (82)	137–139	38.81 (38.53)	5.21 (5.34)	2.52 (3.03)	12.76 (12.34)	20.58 (20.94)	103
[Ni(HL)(H_2_O)_3_Cl]Cl·2H_2_O (C_18_H_27_NO_6_Cl_2_NiFe)	Brown (86)	>300	40.04 (40.78)	5.00 (4.75)	2.59 (2.86)	13.16 (13.67)	21.41 (21.02)	66
[Cu(HL)(H_2_O)_2_Cl_2_]·2H_2_O (C_18_H_25_NO_5_Cl_2_CuFe)	Brown (87)	>300	41.06 (41.58)	4.75 (5.21)	2.66 (2.94)	13.50 (13.22)	22.81 (22.09)	22
[Zn(HL)Cl_2_]·2H_2_O (C_18_H_21_NO_3_Cl_2_ZnFe)	Black (75)	143–145	43.95 (44.56)	4.27 (4.72)	2.85 (2.03)	14.45 (14.14)	24.72 (24.14)	19
[Cd(HL)(H_2_O)_2_Cl_2_] (C_18_H_21_NO_3_Cl_2_CdFe)	Brown (88)	208–210	40.11 (40.87)	3.90 (3.48)	2.60 (2.72)	13.18 (13.76)	31.29 (31.54)	5

**Table 2:** IR spectra (4000–400 cm^−1^) of organometallic acetylferrocene azomethine ligand HL and its metal chelates.

HL	CrHL	MnHL	FeHL	CoHL	NiHL	CuHL	ZnHL	CdHL	Assignment
3444 br	3428 br	3428 br	3415 br	3423 br	3416 br	3417 br	3446 br	3411 br	υ(OH)
1655 sh	1620 m	1626 m	1604 m	1612 m	1624 m	1597 m	1614 sh	1649 m	υ(C=N)
1277 w	1267 w	1281 w	1282 w	1283 w	1277 w	1270 w	1282 w	1281 w	υ(C-O)
------	970 s, 874 w	960 s, 815 w	970 s, 831 w	905 s, 824 s	960 s, 820 w	980 s, 853 w	-----------	894 w, 830 m	υ(H_2_O)
-------	604 w	610 s	620 s	613 m	621 w	681 w	-----------	617 sh	M-O stretch of coordinated water
-------	520 s	598 w	597 w	531 w	533 s	584 s	528 s	534 w	M-O
------	450 s	492 s	472 w	466 s	499 s	460 s	486 w	490 w	M-N

sh = sharp, br = broad, s = small, w = weak, m = medium.

**Table 4:** Thermo-analytical results (TG and DTG) of the organometallic acetylferrocene azomethine ligand (HL) and its metal chelates.

Complex	TG Range (°C)	DTG_max_ (°C)	n*	Mass Loss Total Mass Loss Found (Calcd) %	Assignment	Residues
HL	35–260	198	1	57.13 (57.28)	- Disposal of C_13_H_13_N	FeO
	260–695	363, 689	2	20.53 (20.03) 77.66 (77.31)	- Disposal of C_5_H_4_.	
CrHL	30–140	77	1	9.17 (9.22)	- Disposal of 3H_2_O.	½Cr_2_O_3_ + FeO + 7C
	140–1000	854	1	50.72 (51.11) 59.88 (60.32)	- Disposal of 3HCl, H_2_O and C_11_H_18_NO_0.5_.	
MnHL	45–170	129	1	7.75 (6.54)	- Disposal of H_2_O and NH_3_.	MnO + FeO
	170–440	179, 254, 411	3	10.26 (10.08)	- Disposal of 3H_2_O.	
	440–685	613	1	19.23 (18.86)	- Disposal of 2HCl and C_2_H_4_.	
	685–1000	967	1	35.95 (37.72) 73.19 (73.20)	- Disposal of C_16_H_10_.	
FeHL	50–275	100	1	13.80 (12.40)	- Disposal of 3H_2_O and NH_3_.	½Fe_2_O_3_ + FeO + 12C
	275–580	350	1	15.90 (15.55)	- Disposal of 2HCl and CH_4_.	
	580–1000	634, 666	2	20.21 (20.35) 49.91 (48.12)	- Disposal of C_5_H_12_ClO_0.5_.	
CoHL	45–190	129	1	12.52 (12.40)	- Disposal of 2H_2_O, NH_3_ and CH_4_.	CoO + FeO + 4C
	190–1000	346, 587	2	51.94 (52.65) 64.45 (65.05)	- Disposal of 3H_2_O and C_13_H_12_Cl_2_.	
NiHL	35–145	81	1	9.36 (9.88)	- Disposal of 2H_2_O and NH_3_.	NiO + FeO + 6C
	145–355	224	1	16.60 (16.40)	- Disposal of 2H_2_O, HCl and CH_4_.	
	335–1000	410, 865, 968	3	33.97 (33.09) 59.90 (59.31)	- Disposal of C_11_H_11_Cl.	
CuHL	45–130	91	1	5.78 (6.84)	- Disposal of 2H_2_O.	CuO + FeO + 7C
	130–1000	161, 645	2	48.38 (48.29) 54.15 (55.13)	- Disposal of H_2_O and C_11_H_19_NCl_2_.	
ZnHL	45–275	77	1	6.69 (6.92)	- Disposal of H_2_O and CH_4_.	ZnO + FeO + 3C
	275–730	402, 585	2	24.06 (24.01)	- Disposal of C_2_H_4_, 2HCl and NH_3_.	
	730–1000	954	1	30.82 (30.52) 61.56 (61.44)	- Disposal of C_12_H_6_.	
CdHL	40–220	185	1	27.68 (27.86)	- Disposal of NH_3_, H_2_O, C_3_H_6_ and 2HCl.	CdO + FeO
	220–600	236, 266, 519	3	35.18 (34.91) 62.86 (62.77)	- Disposal of C_15_H_8_.	

n* = number of decomposition stages.

## Data Availability

The raw/processed data generated in this work are available upon request from the corresponding author.

## Supplementary Materials

The following supporting information can be downloaded at: https://www.mdpi.com/article/10.3390/ma15144842/s1, Solutions; Solution of anticancer study; Instruments; Biological activity; Table S1: The different optimized parameters of the free Schiff base ligand; Table S2: The bond lengths and angles of CoHL complex; Table S3: Biological activity of organometallic Schiff base (HL) and its metal complexes; Table S4: Anticancer activity of Schiff base ligand and its metal complexes; Table S5: Energy values obtained in docking calculations with the receptors of (3hb5); Figure S1: FT-IR spectra of (a) HL, (b) Cr(III), (c) Mn(II), (d) Fe(III), (e) Co(II), (f) Ni(II), (g) Cu(II), (h) Zn(II) and (i) Cd(II) complexes; Figure S2: Mass spectrometry of the free acetyl ferrocene azomethine ligand; Figure S3: 1HNMR spectra of (a) HL and (b) its Cd(II) complex Figure S4: The optimized structure of the newly synthesized (A) Schiff base HL and its (B) CoHL complex; Figure S5: Thermal analyses (TG and DTG) of (a) HL2, (b) Cr(III), (c) Mn(II), (d) Fe(III), (e) Co(II), (f) Ni(II), (g) Cu(II), (h) Zn(II) and (i) Cd(II) complexes; Figure S6: Activity index of HL and its metal complexes.
