# Supplementary material for: Design, Structural Inspection and Bio-Medicinal Applications of Some Novel Imine Metal Complexes Based on Acetylferrocene
Source: Materials (Basel). 2022 Jul 12;15(14):4842. doi: 10.3390/ma15144842 (PMC9317992; doi:10.3390/ma15144842)
Supplement: Supplementary file 1 [file materials-15-04842-s001.zip › materials-1765452-supplementary.pdf]

Solutions

Stock solutions of metal complexes of $1x10^{-3}$ M were prepared by dissolving an accurately weighed amount of the complex in DMF. Solution of the Schiff base ligand ($1x10^{-4}$ M) and its metal complexes ($1x10^{-5}$ M) were prepared by dilution of the previous prepared stock solutions for measuring their UV–Vis spectra.

Solution of anticancer study

A fresh stock solution of ($1x10^{-3}$ M) of Schiff base ligand (0.0032 g/L) was prepared in the appropriate volume of ethanol (95 %). Dimethylsulphoxide (DMSO) (Sigma Chemical Co., St. Louis, MO, USA) was used in the cryopreservation of cells. RPMI-1640 medium (Sigma Chemical Co., St. Louis, MO, USA) was used. The medium was used to culture and maintain the human tumor cell line. The medium was supplied in a powder form. It was prepared as follows: 10.4 g medium was weighed, mixed with 2 g sodium bicarbonate, and distilled water was added to 1 L, and the mixture was shaken carefully until complete dissolution. The medium was then sterilized by filtration in a Millipore bacterial filter (0.22 μm). The prepared medium was kept in a refrigerator (4 ºC) and checked at regular intervals for contamination. Before use, the medium was warmed at 37 ºC in a water bath and supplemented with penicillin/streptomycin and FBS. Sodium bicarbonate (Sigma Chemical Co., St. Louis, MO, USA) was used for the preparation of RPMI-1640 medium. Isotonic Trypan blue solution (0.05 %; Sigma Chemical Co., St. Louis, MO, USA) was prepared in normal saline and was used for viability counting. Fetal bovine serum (10 %; FBS) (heat inactivated at 56 ºC for 30 min), 100 units/mL penicillin and 2 mg/mL streptomycin were supplied from Sigma Chemical Co., St. Louis, MO, USA and were used for the supplementation of RPMI-1640 medium prior to use. Trypsin (0.025 % (w/v); Sigma Chemical Co., St. Louis, MO, USA) was used for the harvesting of cells. Acetic acid (1 % (v/v); Sigma Chemical Co., St. Louis, MO, USA) was used for dissolving the unbound SRB dye. Sulphorhodamine-B (0.4 %; SRB) (Sigma Chemical Co., St. Louis, MO, USA) dissolved in 1 % acetic acid was used as a protein dye. A stock solution of trichloroacetic acid (TCA, 50 %, Sigma Chemical Co., St. Louis, MO, USA) was prepared and stored. Fifty microliters of the stock were added to 200 μL RPMI-1640 medium/well to yield a final concentration of 10% used for protein precipitation. One hundred percent isopropanol and 70% ethanol were used. Tris base 10 mM (pH 10.5)

was used for SRB dye solubilization. Tris base (121.1 g) was dissolved in 1000 mL of distilled water, and the pH was adjusted with HCl acid (2 M).

Instruments

Mass spectra were recorded by the EI technique at 70 eV using an MS-5988 GS–MS Hewlett–Packard instrument at the Microanalytical Center, National Center for Research, Egypt. Molar conductivities of 10^{-3} M solutions of the solid complexes in DMF were measured using a Jenway 4010 conductivity meter. Microanalyses of carbon, hydrogen, nitrogen and halogen were carried out at the Microanalytical Center, Cairo University, Egypt, using a CHNS-932 (LECO) Vario Elemental Analyzer. Analyses of the metals followed the dissolution of the solid complexes in concentrated HNO_3, neutralizing the diluted aqueous solutions with ammonia and titrating the metal solutions with EDTA (eriochrome black T was used as indicator for Zn(II), Cd(II) and Mn(II) at pH 10, murexide indicator was used for Co(II), Ni(II) and Cu(II) at pH 12, salicylic acid for Fe(III) at pH <4 and K_2CrO_4 for Cr(III) at pH 5). FT-IR spectra were recorded on a PerkinElmer 1650 spectrometer (4000–400 cm^{-1}) as KBr pellets. Electronic spectra were recorded at room temperature on a Shimadzu 3101pc spectrophotometer as solutions in ethanol. UV–Vis spectra were carried out on UV mini-1240, UV–Vis spectrophotometer, Shimadzu. The thermogravimetric analyses (TG and DTG) of the solid complexes were carried out from room temperature to 1000 ^{o}C using a Shimadzu TG-50H thermal analyzer. The scanning electron microscopic (SEM) image of the complexes was recorded by using SEM model Quanta 250 FEG (Field Emission Gun) attached with EDX Unit (Energy Dispersive X-ray Analyses), with accelerating voltage 30 K.V., magnification 14x up to 1000000 and resolution for Gun.1n, National Research Center, Egypt. The antimicrobial activities were carried out at the Microanalytical Center, Cairo University, Egypt. The anticancer activity was performed at the National Cancer Institute, Cancer Biology Department, Pharmacology Department, Cairo University. The optical density (O.D.) of each well was measured spectrophotometrically at 564 nm with an ELIZA microplate reader (Meter tech. R 960, USA).

Biological activity

Anti-pathogenic activity

The Well diffusion approach was used to test in vitro antifungal and antibacterial activities of ferrocene imine ligand and its metal chelates. DMSO has been used to prepare the stock solutions of different concentrations (µg/mL) to evaluate antimicrobial efficiency. The medium for antifungal activity was prepared by adding 65 g Sabround Dextrose Agar (SDA) in 1 L of distilled water (DW). The mixture of SDA and DW was heated with stirring to form a uniform solution. The sterilization is performed by autoclaving this solution (media) for 15 min at 121 ºC. The media were spread uniformly on sterile Petri plates having a diameter of 90 mm and kept for rest until solidify. The same procedure was followed to prepare nutrient agar (NA) medium i.e., 28 g NA in 1 L of DW to explore antibacterial activity. After solidification, four wells (4 mm diameter) were taken out through sterile cork from the solidified media. The suspension form of the tested fungi was spread over the surface and the separate wells were filled with different concentration stock solutions. Finally, the Petri plates were sealed and stored at low temperature for 2 to 3 h for diffusion and then incubated at room temperature (27 ºC) for 24 h, then the zones of inhibition were evaluated. (mm).

Optimization of anticancer study

To assess the cytotoxicity of the prepared compounds, MCF-7 cell line was incubated in DMEM medium containing FBS (10 percent v/v), streptomycin (100 g/ml), and penicillin (100 g/ml) and maintained at 37°C in a 5 percent CO_2 incubator [33, 35]. Following the incubation period, serial dilutions of the compounds were added to the wells in triplicates, and the incubation period was extended for another 48 hours. Before 1 hour from the end of the incubation, 20 µL of MTT (5 g/mL in PBS) was applied to each well. The plates were shaken after the incubation time and the supernatant liquid was extracted. Each well received 100 µL of DMSO. After 15 minutes of room temperature incubation, the optical density (OD) was measured at 540 nm. The below formula was used to calculate the percentage of cells that were dead.

$$\%\text{Inhibition} = \{(Abs_{control} - Abs_{sample})/(Abs_{sample})\} \times 100$$

The IC_{50} values of the compounds were generated from the dose-response curves and reported as the average of three independent experiments. The IC50 value is determined as the average standard deviation.

Table S1. The different optimized parameters of the free Schiff base ligand.

Atoms	Bond Length	Atoms	Bond Length
C(1)-N(2)	1.3026	C(18)-C(21)	1.4387
C(1)-C(12)	1.5242	C(18)-H(22)	1.0807
C(1)-C(16)	1.4771	C(18)-Fe(25)	2.1127
N(2)-C(3)	1.4147	C(19)-C(21)	1.445
C(3)-C(4)	1.4137	C(19)-H(23)	1.0809
C(3)-C(5)	1.4182	C(19)-Fe(25)	2.1248
C(4)-C(6)	1.4042	C(21)-H(24)	1.0809
C(4)-H(7)	1.0857	C(21)-Fe(25)	2.1199
C(5)-C(8)	1.4022	Fe(25)-C(26)	2.1196
C(5)-H(38)	1.0854	Fe(25)-C(27)	2.1219
C(6)-C(9)	1.408	Fe(25)-C(28)	2.1174
C(6)-O(36)	1.404	Fe(25)-C(30)	2.1232
C(8)-C(9)	1.4097	Fe(25)-C(32)	2.1202
C(8)-H(10)	1.0874	C(26)-C(27)	1.4433
C(9)-H(11)	1.0888	C(26)-C(28)	1.443
C(12)-H(13)	1.0921	C(26)-H(29)	1.0812
C(12)-H(14)	1.0981	C(27)-C(30)	1.4434
C(12)-H(15)	1.0973	C(27)-H(31)	1.0812
C(16)-C(17)	1.448	C(28)-C(32)	1.4436
C(16)-C(18)	1.4515	C(28)-H(33)	1.0811
C(16)-Fe(25)	2.1216	C(30)-C(32)	1.4423
C(17)-C(19)	1.4362	C(30)-H(34)	1.0812
C(17)-H(20)	1.0795	C(32)-H(35)	1.0808
C(17)-Fe(25)	2.1196	O(36)-H(37)	0.9788

Atoms	Bond angle	Atoms	Bond angle

N(2)-C(1)-C(12)	125.5143	C(16)-Fe(25)-C(28)	157.7544
N(2)-C(1)-C(16)	117.5251	C(16)-Fe(25)-C(30)	110.1779
C(12)-C(1)-C(16)	116.9147	C(16)-Fe(25)-C(32)	123.494
C(1)-N(2)-C(3)	125.7581	C(17)-Fe(25)-C(18)	67.0664
N(2)-C(3)-C(4)	122.0017	C(17)-Fe(25)-C(21)	66.7911
N(2)-C(3)-C(5)	118.4063	C(17)-Fe(25)-C(26)	157.0274
C(4)-C(3)-C(5)	119.3687	C(17)-Fe(25)-C(27)	161.6025
C(3)-C(4)-C(6)	119.8155	C(17)-Fe(25)-H(28)	122.3335
C(3)-C(4)-H(7)	121.2474	C(17)-Fe(25)-C(30)	125.5592
C(6)-C(4)-H(7)	118.9161	C(17)-Fe(25)-C(32)	108.9573
C(3)-C(5)-C(8)	119.8965	C(18)-Fe(25)-C(19)	66.8211
C(3)-C(5)-H(38)	118.8807	C(18)-Fe(25)-C(26)	125.0584
C(8)-C(5)-H(38)	121.2219	C(18)-Fe(25)-C(27)	109.9798
C(4)-C(6)-C(9)	121.2318	C(18)-Fe(25)-C(28)	160.1306
C(4)-C(6)-O(36)	116.3428	C(18)-Fe(25)-C(30)	124.3456
C(9)-C(6)-O(36)	122.4252	C(18)-Fe(25)-C(32)	158.9599
C(5)-C(8)-C(9)	121.0919	C(19)-Fe(25)-C(26)	122.4934

C(5)-C(8)-H(10)	119.5852	C(19)-Fe(25)-C(27)	157.8217
C(9)-C(8)-H(10)	119.3193	C(19)-Fe(25)-C(28)	108.218
C(6)-C(9)-C(8)	118.5768	C(19)-Fe(25)-C(30)	160.406
C(6)-C(9)-H(11)	120.7983	C(19)-Fe(25)-C(32)	124.2824
C(8)-C(9)-H(11)	120.6211	C(21)-Fe(25)-C(26)	108.7996
C(1)-C(12)-H(13)	111.7997	C(21)-Fe(25)-C(27)	123.4916
C(1)-C(12)-H(14)	110.6569	C(21)-Fe(25)-C(28)	124.1332
C(1)-C(12)-H(15)	109.7583	C(21)-Fe(25)-C(30)	158.812
H(13)-C(12)-H(14)	108.5607	C(21)-Fe(25)-C(32)	159.7751
H(13)-C(12)-H(15)	108.6827	C(26)-Fe(25)-C(30)	66.7661
H(14)-C(12)-H(15)	107.2518	C(26)-Fe(25)-C(32)	66.8655
C(1)-C(16)-C(17)	125.2937	C(27)-Fe(25)-C(28)	66.8408
C(1)-C(16)-C(18)	127.2251	C(27)-Fe(25)-C(32)	66.8231
C(1)-C(16)-Fe(25)	125.3787	C(28)-Fe(25)-C(30)	66.7764
C(17)-C(16)-C(18)	107.4796	Fe(25)-C(26)-H(29)	124.918
C(16)-C(17)-C(19)	108.2255	C(27)-C(26)-C(28)	107.9915
C(16)-C(17)-H(20)	124.2547	C(27)-C(26)-H(29)	126.012
C(19)-C(17)-H(20)	127.5171	C(28)-C(26)-H(29)	125.9937

H(20)-C(17)-Fe(25)	125.616	Fe(25)-C(27)-H(31)	125.5143
C(16)-C(18)-C(21)	108.1004	C(26)-C(27)-C(30)	107.9424
C(16)-C(18)-H(22)	126.3206	C(26)-C(27)-H(31)	125.9386
C(21)-C(18)-H(22)	125.5779	C(30)-C(27)-H(31)	126.1187
H(22)-C(18)-Fe(25)	125.2532	Fe(25)-C(28)-H(33)	124.7203
C(17)-C(19)-C(21)	108.166	C(26)-C(28)-C(32)	108.0433
C(17)-C(19)-H(23)	125.9701	C(26)-C(28)-H(33)	125.9545
C(21)-C(19)-H(23)	125.8614	C(32)-C(28)-H(33)	125.9986
H(23)-C(19)-Fe(25)	125.2752	Fe(25)-C(30)-H(34)	125.334
C(18)-C(21)-C(19)	108.0251	C(27)-C(30)-C(32)	108.0942
C(18)-C(21)-H(24)	125.9227	C(27)-C(30)-H(34)	126.1627
C(19)-C(21)-H(24)	126.0521	C(32)-C(30)-H(34)	125.7426
H(24)-C(21)-Fe(25)	125.3964	Fe(25)-C(32)-H(35)	124.8331
C(16)-Fe(25)-C(19)	66.7745	C(28)-C(32)-C(30)	107.9284
C(16)-Fe(25)-C(21)	66.9557	C(28)-C(32)-H(35)	126.1557
C(16)-Fe(25)-C(26)	161.4019	C(30)-C(32)-H(35)	125.9128
C(16)-Fe(25)-C(27)	126.0359	C(6)-O(36)-H(37)	111.8374

Table S2. The bond lengths and angles of CoHL complex

Bond lengths			
Cl(26)-H(61)	1.336	C(15)-H(36)	1.095
Cl(25)-H(60)	1.336	C(15)-C(16)	1.55
Cl(26)-H(59)	1.312	C(14)-H(35)	1.099
Cl(25)-H(58)	1.356	C(14)-C(15)	1.532
C(17)-H(57)	1.141	C(13)-H(34)	1.104
C(17)-H(56)	1.145	C(13)-C(17)	1.55
C(16)-H(55)	1.099	C(13)-C(14)	1.539
C(15)-H(54)	1.099	C(12)-H(33)	1.096
C(14)-H(53)	1.094	C(11)-C(19)	1.506
C(13)-H(52)	1.097	C(11)-C(12)	1.549
C(12)-H(51)	1.101	C(10)-Fe(18)	2.093
C(11)-H(50)	1.097	C(10)-C(11)	1.576
C(10)-H(49)	1.152	C(9)-H(32)	1.101
C(10)-H(48)	1.161	C(9)-C(10)	1.554
C(9)-H(47)	1.098	C(8)-H(31)	1.1
C(8)-H(46)	1.098	C(8)-C(12)	1.538
O(24)-H(45)	1.005	C(8)-C(9)	1.54
O(24)-H(44)	0.973	N(7)-Co(21)	1.533
O(23)-H(43)	0.965	C(19)-N(7)	1.323
O(23)-H(42)	0.969	C(6)-H(30)	1.087
O(22)-H(41)	0.972	C(5)-H(29)	1.093
Co(21)-Cl(26)	3.488	C(5)-C(6)	1.406
Co(21)-Cl(25)	2.43	C(4)-H(28)	1.091
O(24)-Co(21)	2.265	C(4)-C(5)	1.42
O(23)-Co(21)	2.94	C(3)-O(22)	1.412
O(22)-Co(21)	1.647	C(3)-C(4)	1.382

C(20)-H(40)	1.104	C(2)-H(27)	1.095
C(20)-H(39)	1.109	C(2)-C(3)	1.425
C(20)-H(38)	1.104	C(1)-N(7)	1.412
C(19)-C(20)	1.504	C(6)-C(1)	1.414
C(17)-Fe(18)	2.113	C(1)-C(2)	1.466
C(16)-H(37)	1.099	C(16)-C(17)	1.567
Bond angles			
H(61)-Cl(26)-H(59)	51.184	H(35)-C(14)-C(15)	111.272
H(61)-Cl(26)-Co(21)	24.917	H(35)-C(14)-C(13)	109.83
H(59)-Cl(26)-Co(21)	68.711	C(15)-C(14)-C(13)	103.849
H(60)-Cl(25)-H(58)	81.011	H(52)-C(13)-H(34)	107.135
H(60)-Cl(25)-Co(21)	17.511	H(52)-C(13)-C(17)	111.886
H(58)-Cl(25)-Co(21)	89.232	H(52)-C(13)-C(14)	113.237
H(45)-O(24)-H(44)	102.661	H(34)-C(13)-C(17)	110.35
H(45)-O(24)-Co(21)	107.657	H(34)-C(13)-C(14)	110.515
H(44)-O(24)-Co(21)	107.361	C(17)-C(13)-C(14)	103.753
H(43)-O(23)-H(42)	103.658	H(51)-C(12)-H(33)	107.213
H(43)-O(23)-Co(21)	59.217	H(51)-C(12)-C(11)	110.507
H(42)-O(23)-Co(21)	93.286	H(51)-C(12)-C(8)	109.901
H(41)-O(22)-Co(21)	141.028	H(33)-C(12)-C(11)	111.695
H(41)-O(22)-C(3)	107.91	H(33)-C(12)-C(8)	113.362
Co(21)-O(22)-C(3)	76.12	C(11)-C(12)-C(8)	104.19
Cl(26)-Co(21)-Cl(25)	83.784	H(50)-C(11)-C(19)	105.77
Cl(26)-Co(21)-O(24)	155.789	H(50)-C(11)-C(12)	108.713
Cl(26)-Co(21)-O(23)	73.199	H(50)-C(11)-C(10)	105.897
Cl(26)-Co(21)-O(22)	87.45	C(19)-C(11)-C(12)	116.015
Cl(26)-Co(21)-N(7)	86.796	C(19)-C(11)-C(10)	115.762

Cl(25)-Co(21)-O(24)	92.886	C(12)-C(11)-C(10)	104.153
Cl(25)-Co(21)-O(23)	82.193	H(49)-C(10)-H(48)	113.699
Cl(25)-Co(21)-O(22)	56.337	H(49)-C(10)-Fe(18)	58.35
Cl(25)-Co(21)-N(7)	165.607	H(49)-C(10)-C(11)	111.012
O(24)-Co(21)-O(23)	82.591	H(49)-C(10)-C(9)	109.438
O(24)-Co(21)-O(22)	110.642	H(48)-C(10)-Fe(18)	55.569
O(24)-Co(21)-N(7)	100.112	H(48)-C(10)-C(11)	107.721
O(23)-Co(21)-O(22)	136.124	H(48)-C(10)-C(9)	107.886
O(23)-Co(21)-N(7)	105.46	Fe(18)-C(10)-C(11)	122.853
O(22)-Co(21)-N(7)	112.479	Fe(18)-C(10)-C(9)	130.166
H(40)-C(20)-H(39)	107.321	C(11)-C(10)-C(9)	106.808
H(40)-C(20)-H(38)	108.732	H(47)-C(9)-H(32)	106.265
H(40)-C(20)-C(19)	111.037	H(47)-C(9)-C(10)	112.472
H(39)-C(20)-H(38)	106.071	H(47)-C(9)-C(8)	113.852
H(39)-C(20)-C(19)	113.485	H(32)-C(9)-C(10)	110.051
H(38)-C(20)-C(19)	109.97	H(32)-C(9)-C(8)	109.605
C(20)-C(19)-C(11)	121.542	C(10)-C(9)-C(8)	104.616
C(20)-C(19)-N(7)	121.378	H(46)-C(8)-H(31)	107.505
C(11)-C(19)-N(7)	117.079	H(46)-C(8)-C(12)	112.674
C(17)-Fe(18)-C(10)	177.443	H(46)-C(8)-C(9)	112.467
H(57)-C(17)-H(56)	114.042	H(31)-C(8)-C(12)	109.847
H(57)-C(17)-Fe(18)	58.753	H(31)-C(8)-C(9)	110.925
H(57)-C(17)-C(16)	110.901	C(12)-C(8)-C(9)	103.435
H(57)-C(17)-C(13)	110.747	Co(21)-N(7)-C(19)	147.641
H(56)-C(17)-Fe(18)	55.544	Co(21)-N(7)-C(1)	83.961
H(56)-C(17)-C(16)	106.594	C(19)-N(7)-C(1)	128.378
H(56)-C(17)-C(13)	108.095	H(30)-C(6)-C(5)	119.044

Fe(18)-C(17)-C(16)	130.783	H(30)-C(6)-C(1)	120.955
Fe(18)-C(17)-C(13)	122.837	C(5)-C(6)-C(1)	119.857
C(16)-C(17)-C(13)	106.063	H(29)-C(5)-C(6)	120.683
H(55)-C(16)-H(37)	106.233	H(29)-C(5)-C(4)	119.256
H(55)-C(16)-C(17)	112.836	C(6)-C(5)-C(4)	120.037
H(55)-C(16)-C(15)	111.7	H(28)-C(4)-C(5)	120.975
H(37)-C(16)-C(17)	109.583	H(28)-C(4)-C(3)	119.734
H(37)-C(16)-C(15)	110.84	C(5)-C(4)-C(3)	119.289
C(17)-C(16)-C(15)	105.71	O(22)-C(3)-C(4)	121.372
H(54)-C(15)-H(36)	107.345	O(22)-C(3)-C(2)	114.912
H(54)-C(15)-C(16)	110.132	C(4)-C(3)-C(2)	123.551
H(54)-C(15)-C(14)	109.602	H(27)-C(2)-C(3)	118.892
H(36)-C(15)-C(16)	112.041	H(27)-C(2)-C(1)	120.507
H(36)-C(15)-C(14)	112.756	C(3)-C(2)-C(1)	114.882
C(16)-C(15)-C(14)	104.97	N(7)-C(1)-C(6)	126.178
H(53)-C(14)-H(35)	106.03	N(7)-C(1)-C(2)	111.006
H(53)-C(14)-C(15)	113.066	C(6)-C(1)-C(2)	120.486
H(53)-C(14)-C(13)	112.887		

Table S3: Biological activity of organometallic Schiff base (HL) and its metal complexes.

Compound	Inhibition zone (mm/mg sample)					
	Fungi		Gram-positive bacteria		Gram-negative bacteria	
	Aspergillus fumigatus	*Candida albicans*	*Staphylococcus aureus*	*Bacillus subtilis*	*Salmonella typhimurium*	*Escherichia coli*
HL	NA	NA	NA	NA	NA	NA
$[Cr(HL)(H_2O)_3Cl]Cl_2.3H_2O$	NA	NA	NA	NA	NA	NA
$[Mn(HL)(H_2O)_4]Cl_2.H_2O$	NA	NA	NA	NA	NA	NA
$[Fe(HL)(H_2O)_2Cl_2]Cl.3H_2O$	NA	NA	NA	NA	NA	NA
$[Co(HL)(H_2O)_4]Cl_2.2H_2O$	NA	10	20	18	16	19
$[Ni(HL)(H_2O)_3Cl]Cl.2H_2O$	NA	NA	NA	NA	NA	NA
$[Cu(HL)(H_2O)_2Cl_2].2H_2O$	NA	NA	18	17	13	17
$[Zn(HL)Cl_2].2H_2O$	NA	10	17	16	15	14
$[Cd(HL)(H_2O)_2Cl_2]$	NA	12	18	15	17	16
Control	*Ketokenazole*		*Gentamycin*		*Gentamycin*	
	17	20	24	26	17	30

Table S4. Anticancer activity of Schiff base ligand and its metal complexes.

Complex	Concn. (µg/ ml)	Surviving fraction (MCF7)					IC_{50} (µg/ ml)
		0.0	5.0	12.5	25.0	50.0	
$[Mn(HL)(H_2O)_4]Cl_2.H_2O$		1.00	0.82	0.73	0.64	0.55	36.70
$[Cu(HL)(H_2O)_2Cl_2].2H_2O$		1.00	0.87	0.77	0.71	0.64	36.4
$[Cd(HL)(H_2O)_2Cl_2]$		1.00	1.00	0.74	0.68	0.65	3.5

Table S5. Energy values obtained in docking calculations with the receptors of (3hb5).

Compounds	Ligand moiety	Receptor site	Interaction	Distance (ºA)	*E* (kcal/mol)
3-aminophenol	N 1	O GLY 92 (X)	H-donor	2.95	-2.2
	O 11	O SER 12 (X)	H-donor	2.8	-1.0
2-acetyl ferrocene	O 24	N ILE 14 (X)	H-acceptor	3.08	-1.9
	Fe 14	O GLY 92 (X)	metal	2.32	-2.8
	C 20	NE ARG 37 (X)	ionic	3.84	-0.8
	5-ring	NH2 ARG 37 (X)	pi-cation	3.68	-0.7
HL	N 2	NE ARG 37 (X)	H-acceptor	2.93	-4.6
	N 2	NH2 ARG 37 (X)	H-acceptor	2.91	-0.8
	O 34	N SER 11 (X)	H-acceptor	2.89	-2.0
	C 30	NE ARG 37 (X)	ionic	3.9	-0.7
	C 30	NH1 ARG 37 (X)	ionic	3.87	-0.8
	C 30	NH2 ARG 37 (X)	ionic	3.73	-1.1
$[Mn(HL)(H_2O)_4]Cl_2.H_2O$	O 41	OG SER 142 (X)	H-donor	3.19	-10.0
	O 44	OG SER 142 (X)	H-donor	3.6	-1.1
	O 47	OG SER 142 (X)	H-donor	2.7	-38.7
	O 47	OH TYR 155 (X)	H-donor	2.54	-0.8
	O 41	OG SER 142 (X)	ionic	3.19	-3.3
	O 44	OG SER 142 (X)	ionic	3.6	-1.6
	O 47	OG SER 142 (X)	ionic	2.7	-6.8

	O 47	OH TYR 155 (X)	ionic	2.54	-8.4
$[Cu(HL)(H_2O)_2Cl_2].2H_2O$	O 35	OD1 ASN 152 (X)	H-donor	2.82	-8.9
	O 38	OD1 ASN 152 (X)	H-donor	2.52	-10.8
	O 35	6-ring TYR 155 (X)	cation-pi	3.21	-0.7
$[Cd(HL)(H_2O)_2Cl_2]$	C 8	SG CYS 185 (X)	H-donor	3.76	-0.7
	C 9	O GLY 141 (X)	H-donor	3.41	-0.8
	O 34	OH TYR 155 (X)	H-donor	2.56	-29.4
	O 38	OG SER 142 (X)	H-donor	3.17	0.1
	O 41	OH TYR 155 (X)	H-donor	2.85	-18.5
	O 44	O GLY 186 (X)	H-donor	2.5	-0.8
	Cl 48	O CYS 185 (X)	H-donor	2.9	-0.3
	Fe 24	O VAL 188 (X)	metal	1.88	-4.1
	O 34	OH TYR 155 (X)	ionic	2.56	-8.2
	O 38	OH TYR 155 (X)	ionic	2.93	-5.0
	O 41	OH TYR 155 (X)	ionic	2.85	-5.6

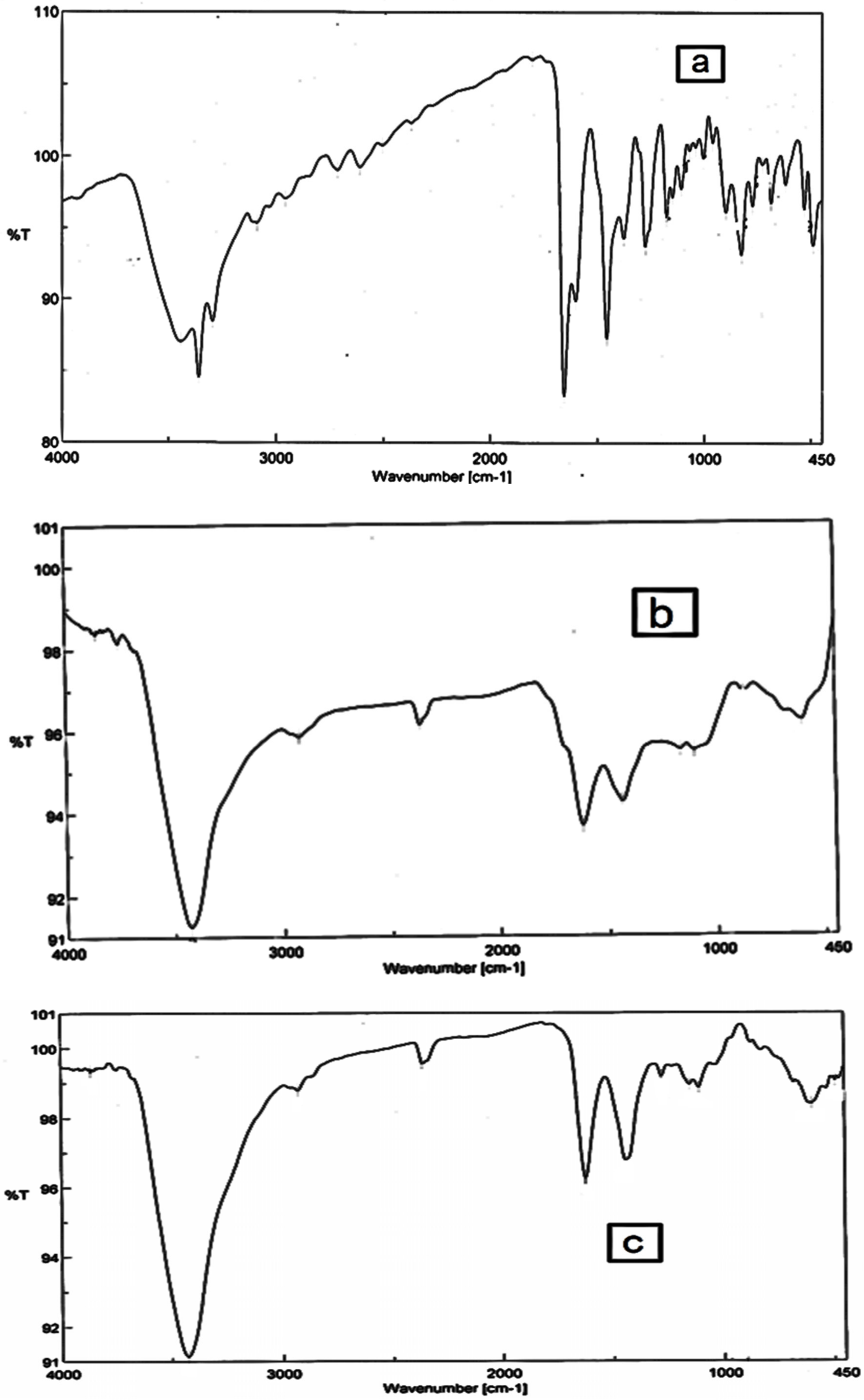
a
110
100
90
80
%T
4000
3000
2000
1000
450
Wavenumber [cm-1]
b
101
100
98
96
94
92
91
%T
4000
3000
2000
1000
450
Wavenumber [cm-1]
c
101
100
98
96
94
92
91
%T
4000
3000
2000
1000
450
Wavenumber [cm-1]

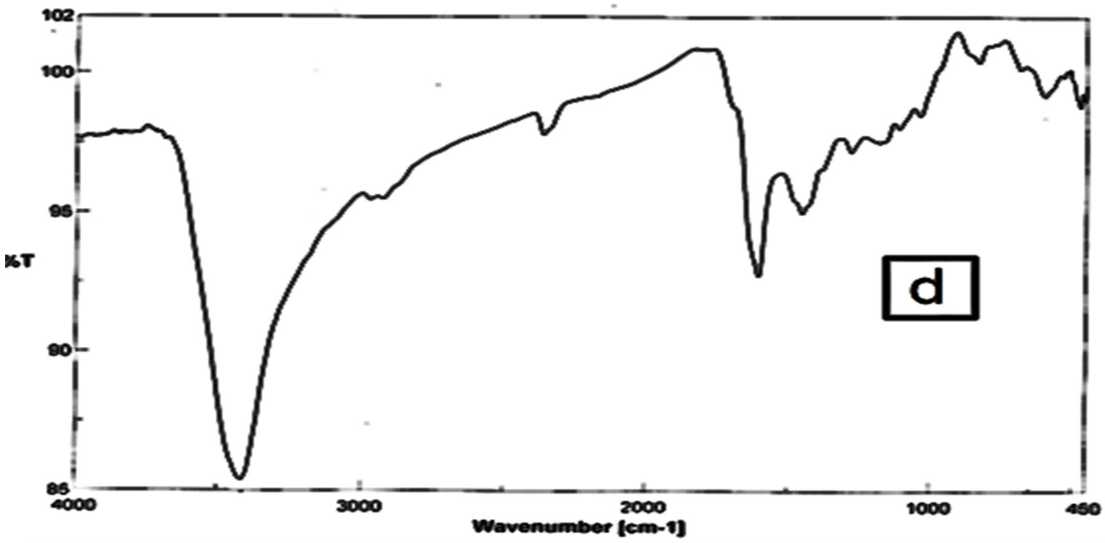
102
100
95
%T
90
85
4000
3000
2000
1000
450
Wavenumber [cm-1]
d

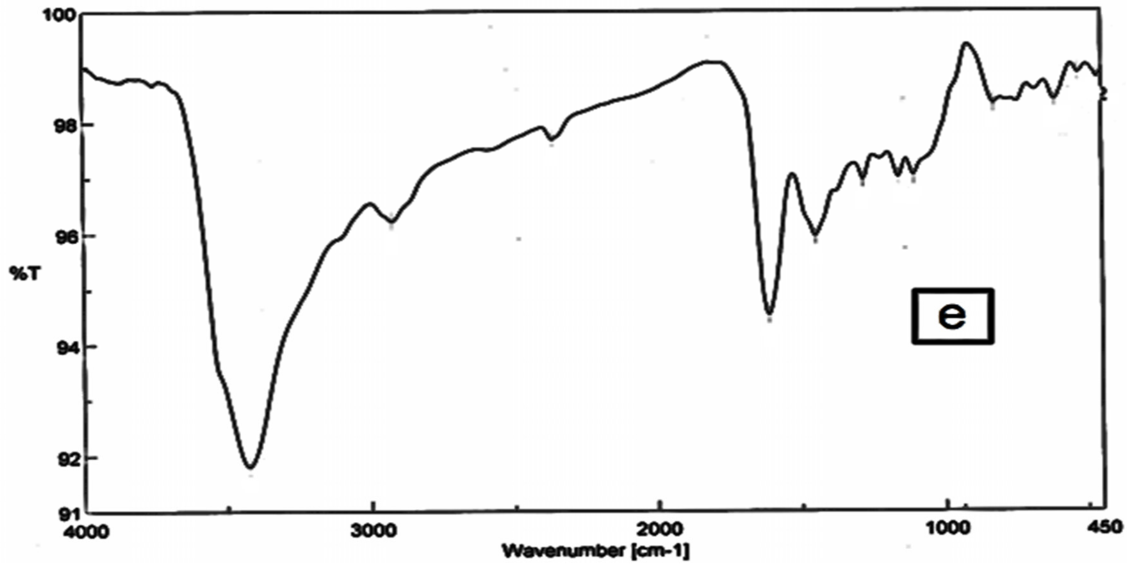
100
98
96
%T
94
92
91
4000
3000
2000
1000
450
Wavenumber [cm-1]
e

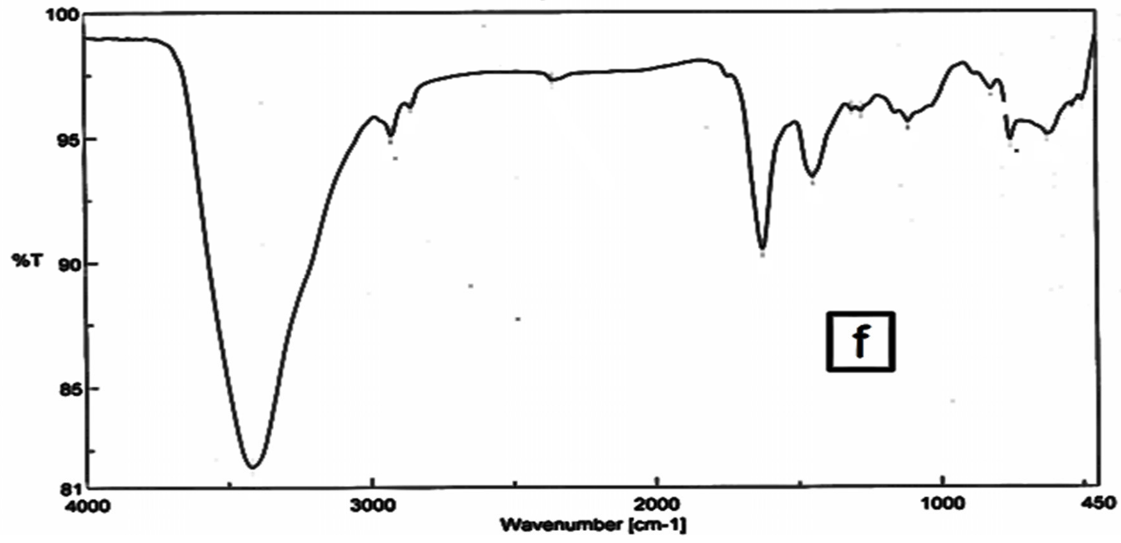
100
95
%T
90
85
81
4000
3000
2000
1000
450
Wavenumber [cm-1]
f

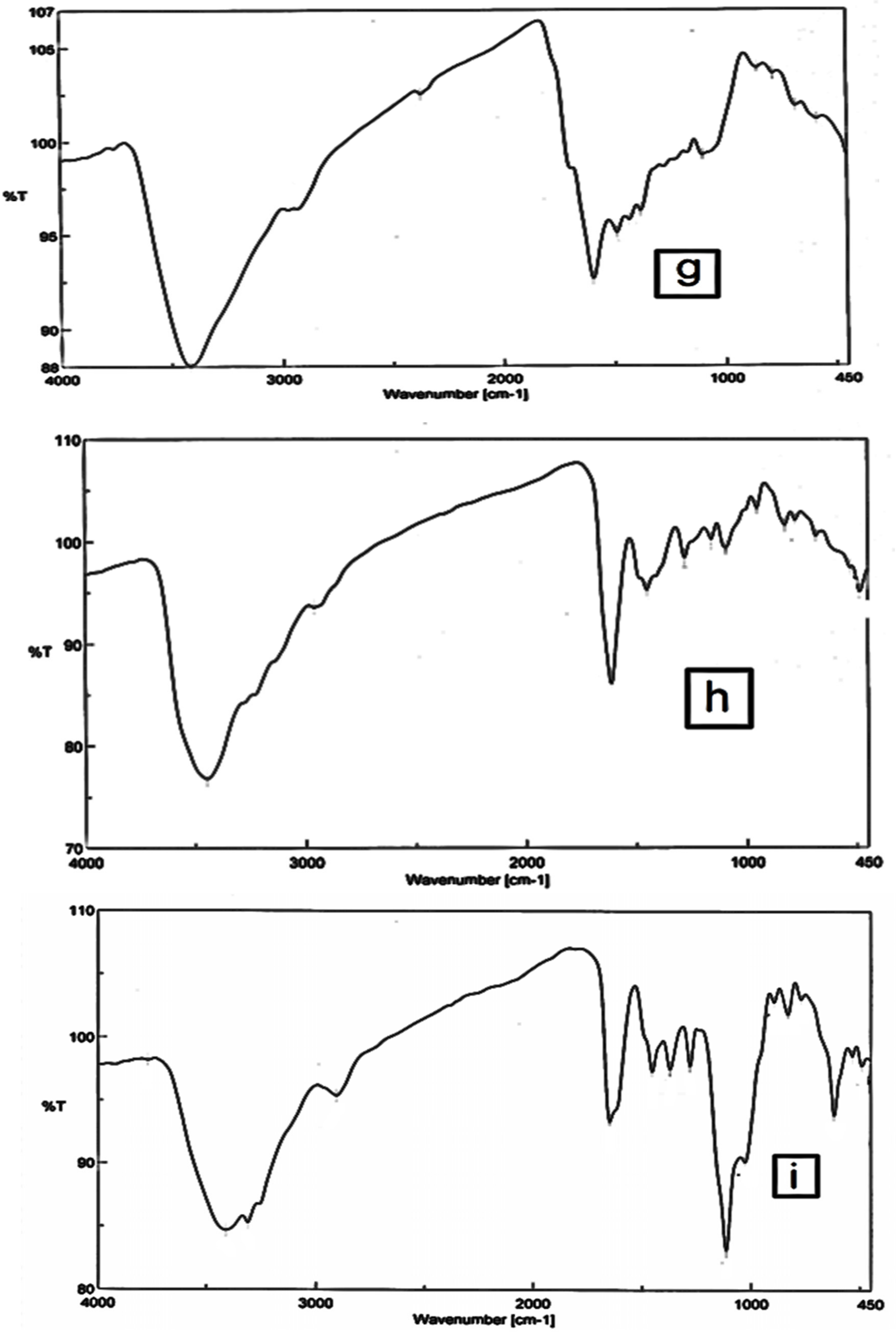

Figure S1. FT-IR spectra of a) HL, b) Cr(III), c) Mn(II), d) Fe(III), e) Co(II), f) Ni(II), g) Cu(II), h) Zn(II) and i) Cd(II) complexes.

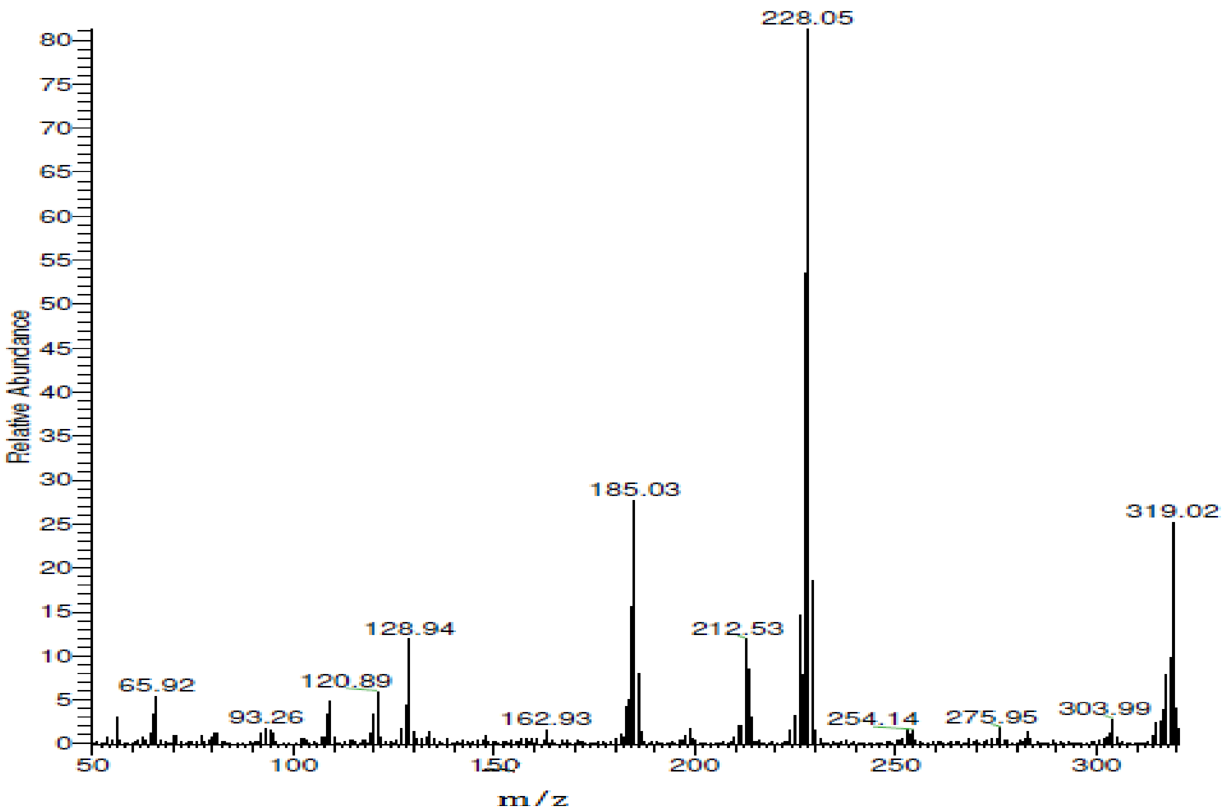

Figure S2. Mass spectrometry of the free acetyl ferrocene azomethine ligand.

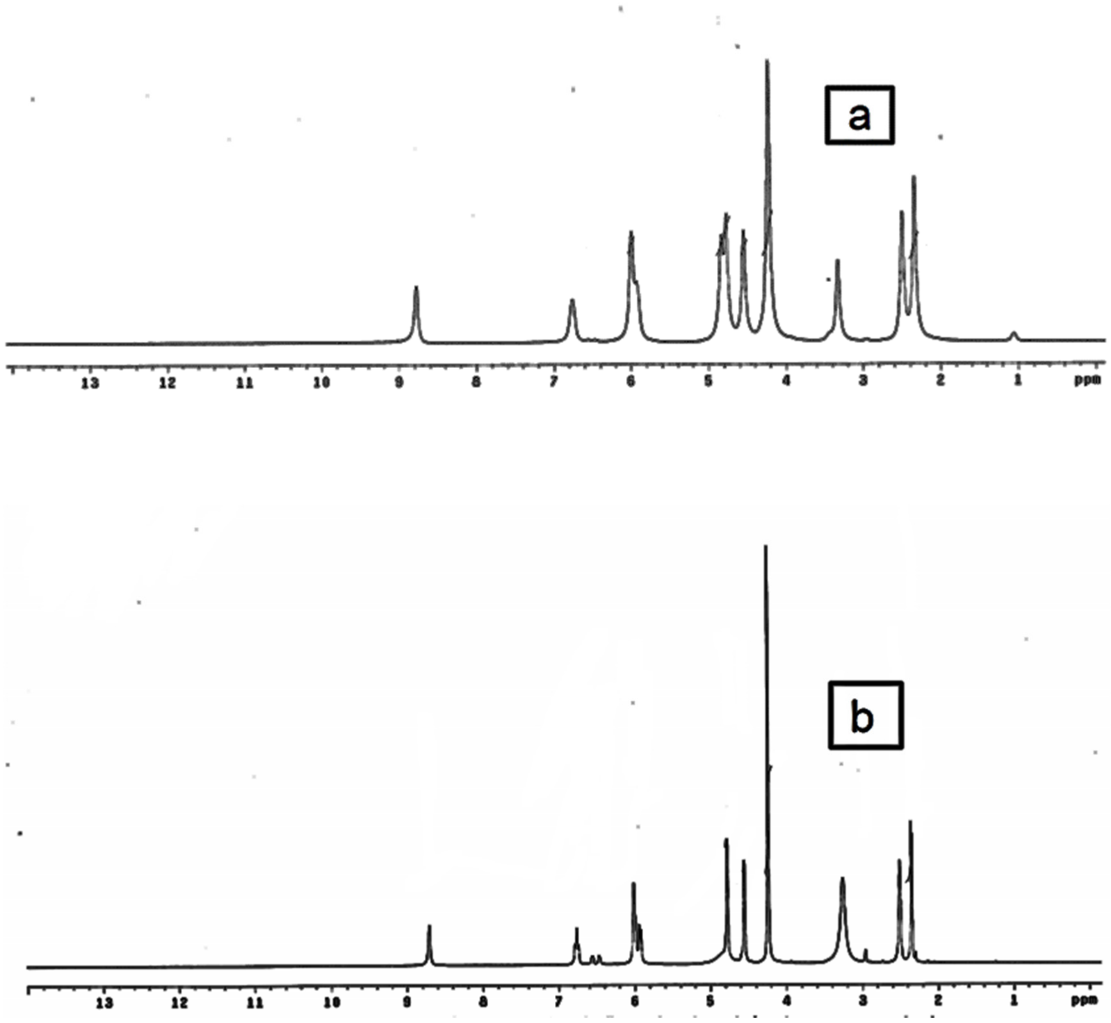

Figure S3. ^{1}HNMR spectra of a) HL and b) its Cd(II) complex.

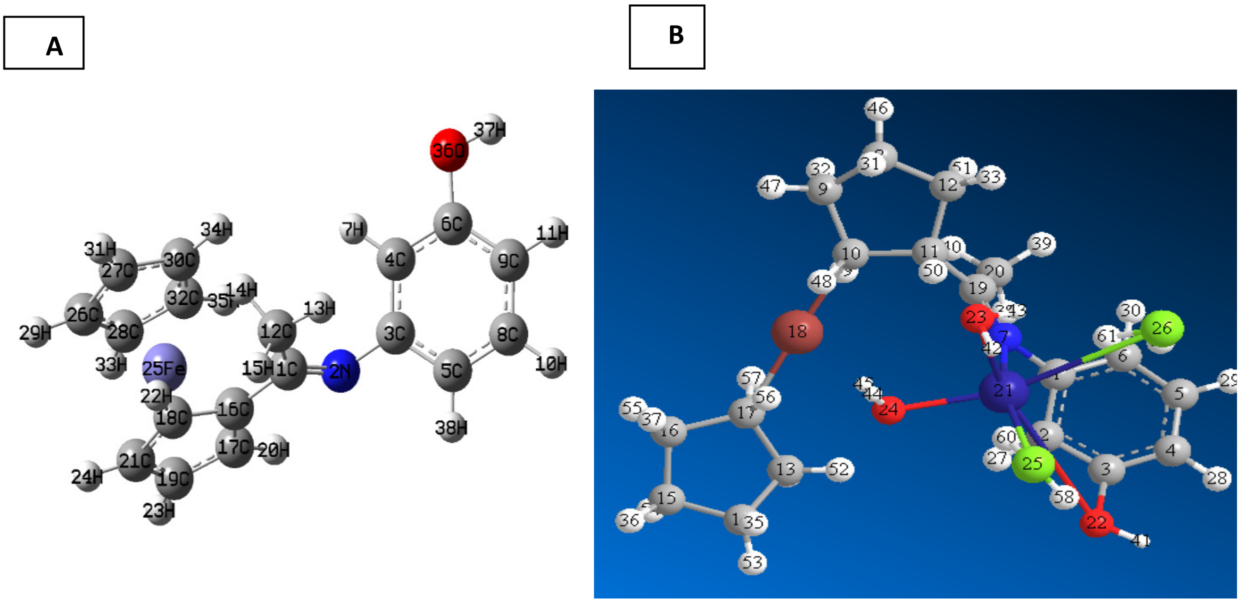

Figure S4. The optimized structure of the newly synthesized A) Schiff base HL and its B) CoHL complex.

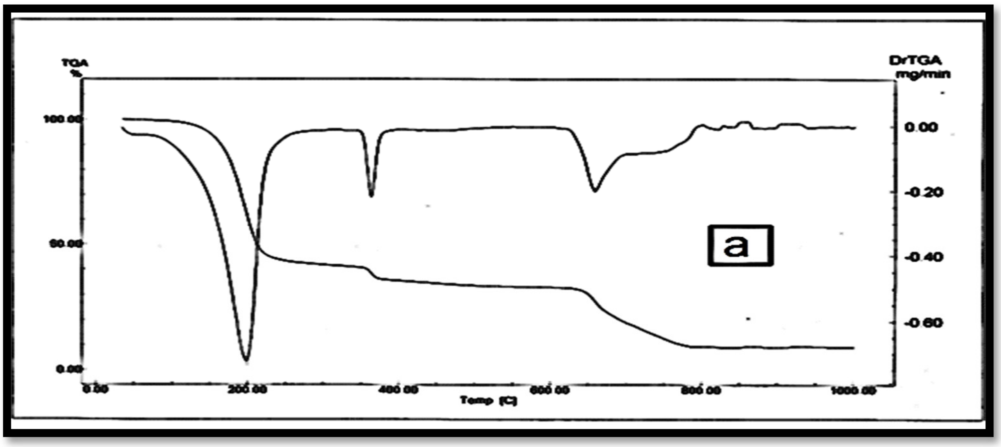
TGA
%
DrTGA
mg/min
100.00
50.00
0.00
0.00
-0.20
-0.40
-0.60
0.00
200.00
400.00
600.00
800.00
1000.00
Temp [C]
a

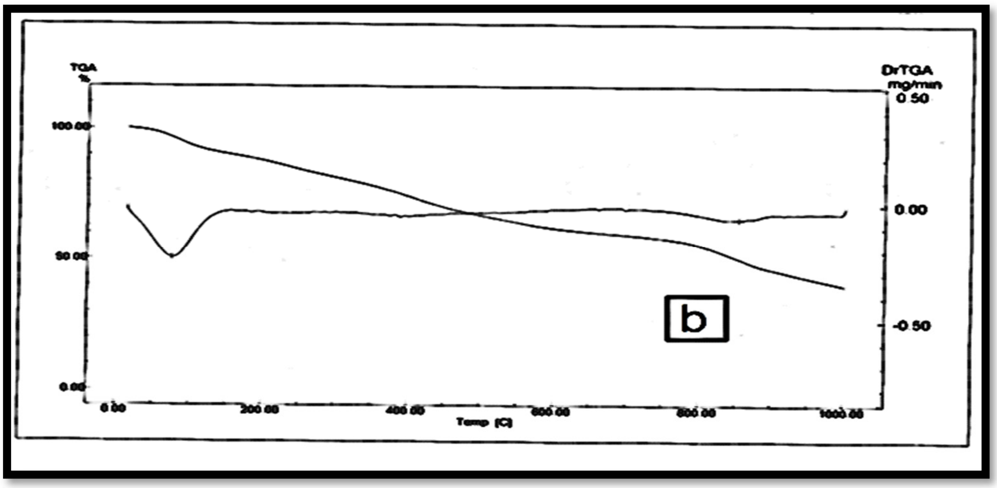
TGA
%
DrTGA
mg/min
100.00
50.00
0.00
0.50
0.00
-0.50
0.00
200.00
400.00
600.00
800.00
1000.00
Temp [C]
b

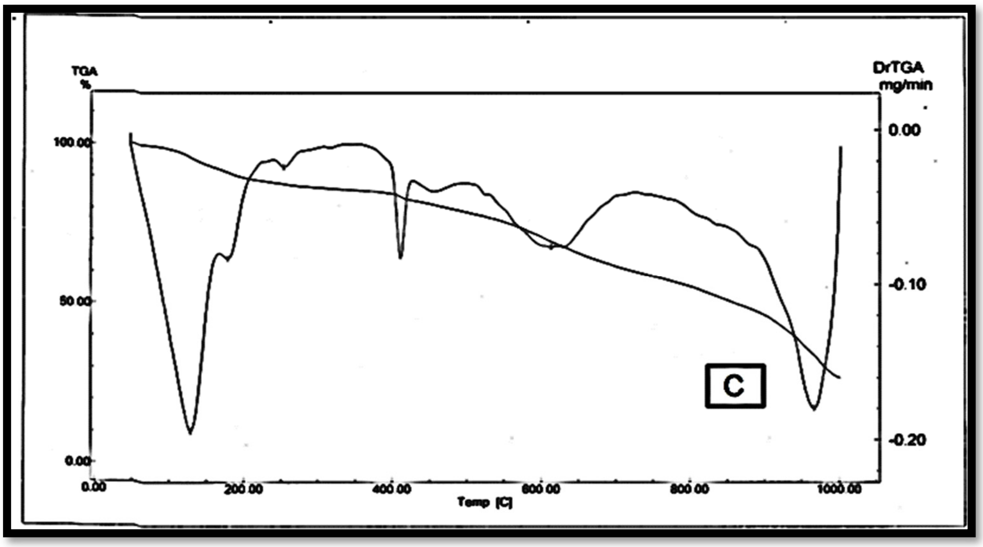
TGA
%
DrTGA
mg/min
100.00
50.00
0.00
0.00
-0.10
-0.20
0.00
200.00
400.00
600.00
800.00
1000.00
Temp [C]
c

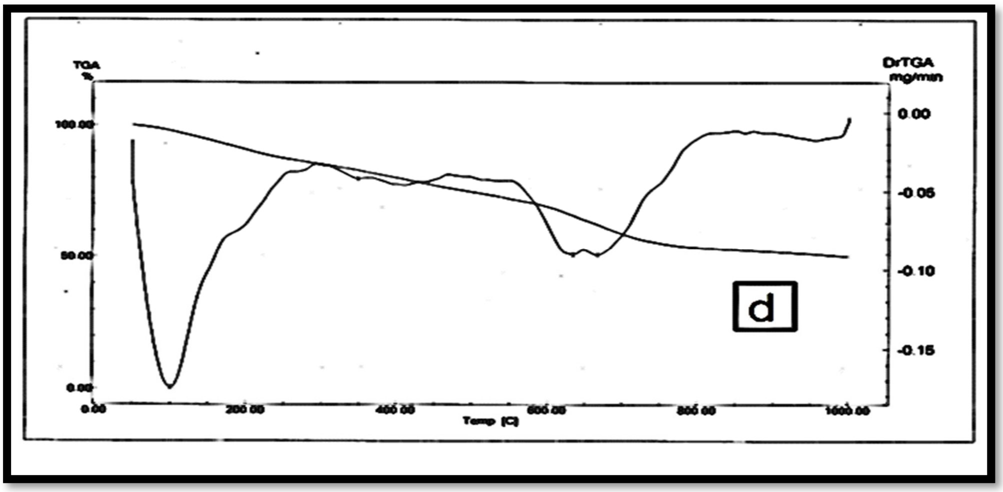
TGA
%
DrTGA
mg/min
100.00
50.00
0.00
0.00
-0.05
-0.10
-0.15
0.00
200.00
400.00
600.00
800.00
1000.00
Temp (C)
d

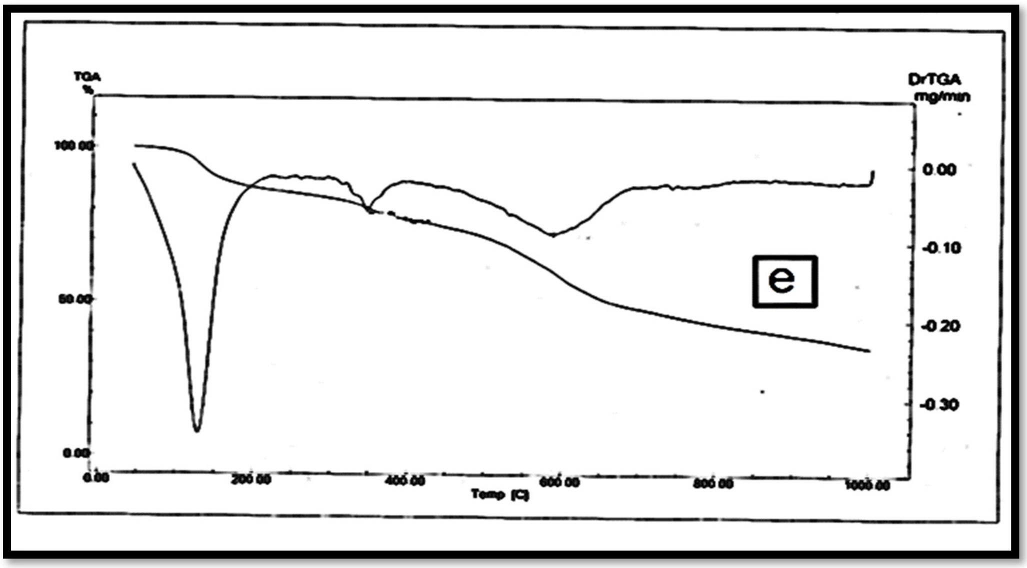
TGA
%
DrTGA
mg/min
100.00
50.00
0.00
0.00
-0.10
-0.20
-0.30
0.00
200.00
400.00
600.00
800.00
1000.00
Temp (C)
e

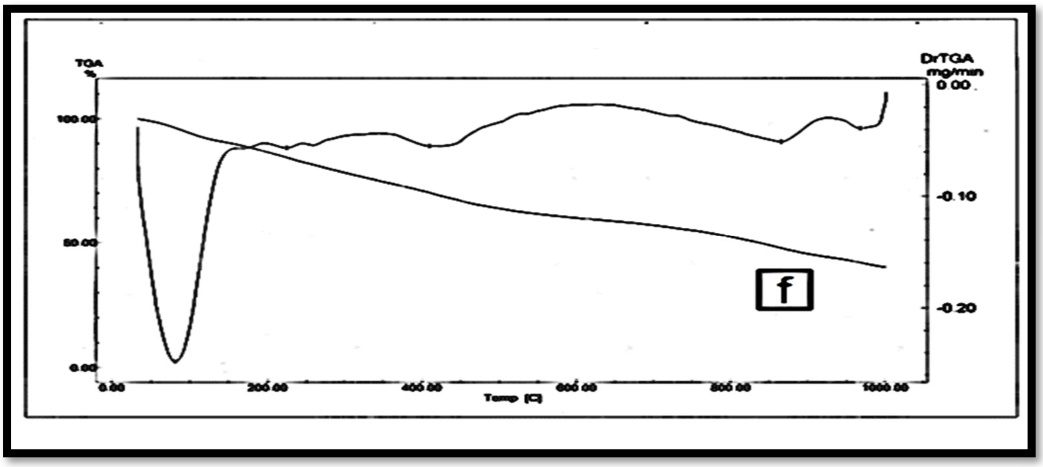
TGA
%
DrTGA
mg/min
100.00
50.00
0.00
0.00
-0.10
-0.20
0.00
200.00
400.00
600.00
800.00
1000.00
Temp (C)
f

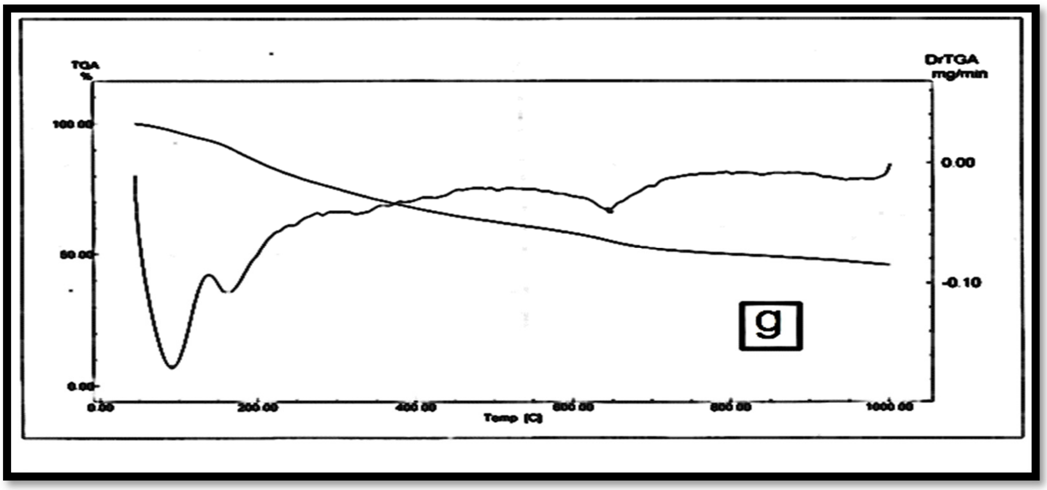

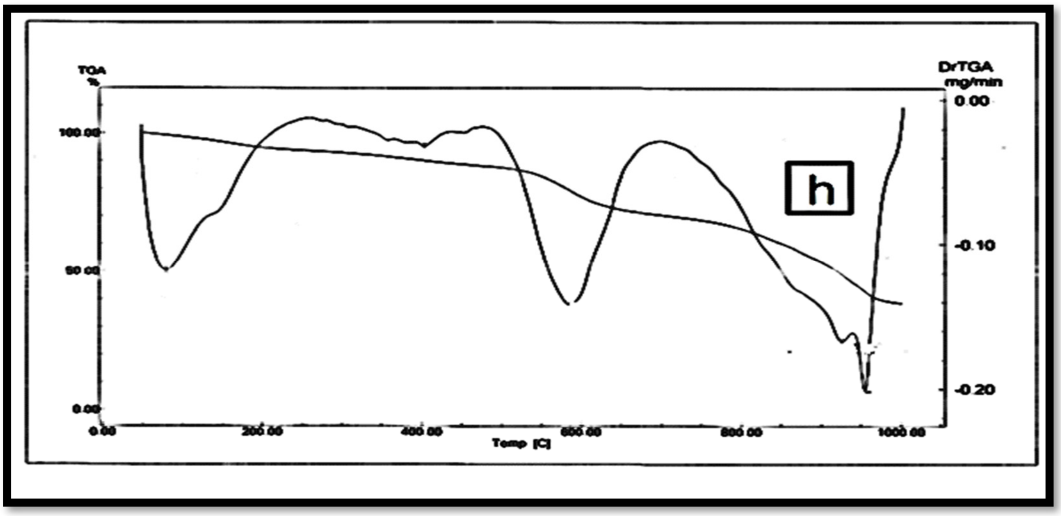

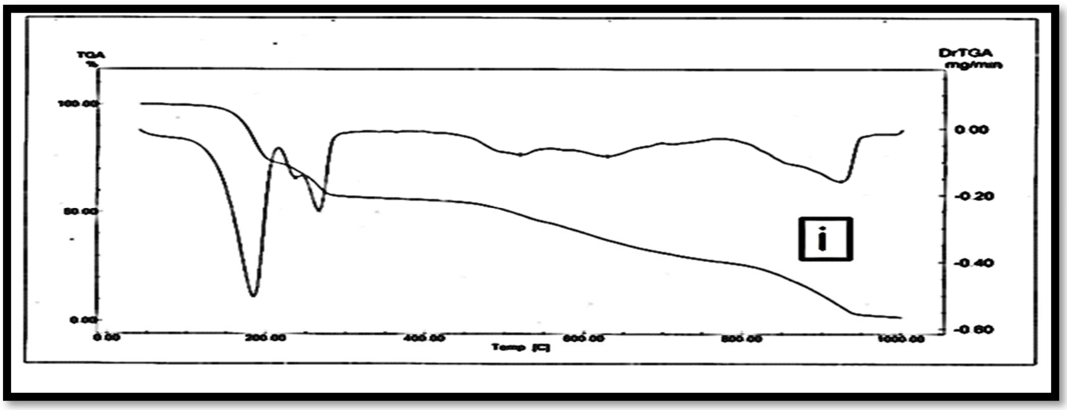

Figure S5. Thermal analyses (TG and DTG) of a) HL^2, b) Cr(III), c) Mn(II), d) Fe(III), e) Co(II), f) Ni(II), g) Cu(II), h) Zn(II) and i) Cd(II) complexes.

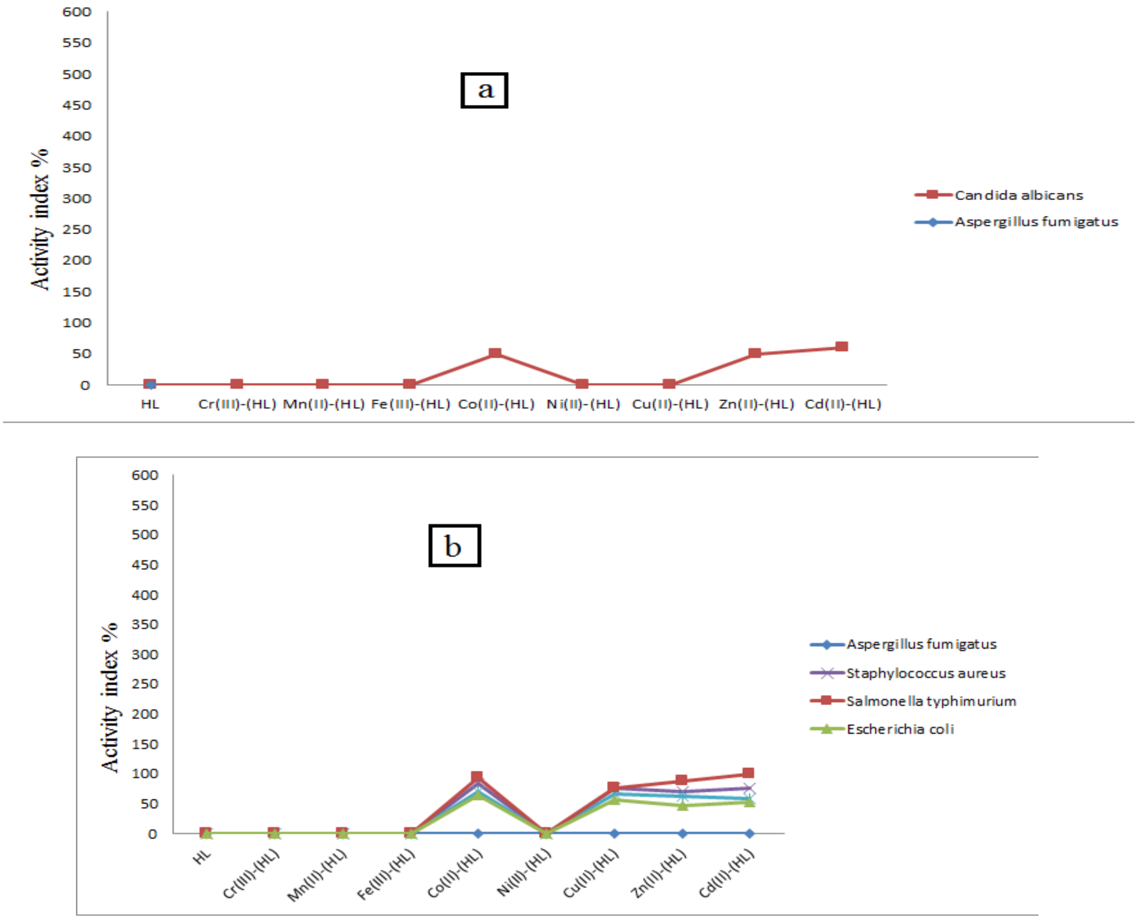

Figure S6. Activity index of HL and its metal complexes.
